# Tumor microenvironment-responsive DNA-based nanomedicine triggers innate sensing for enhanced immunotherapy

**DOI:** 10.1186/s12951-023-02132-6

**Published:** 2023-10-19

**Authors:** Jinyang Li, Xiaoyu Han, Shanshan Gao, Yumeng Yan, Xiaoguang Li, Hui Wang

**Affiliations:** State Key Laboratory of Systems Medicine for Cancer, Center for Single-Cell Omics, School of Public Health, Shanghai Jiao Tong University School of Medicine, Shanghai, 200025 China

**Keywords:** Innate sensing, Tumor immunotherapy, Type I interferon, Immune checkpoint blockade, Nanomedicine

## Abstract

Lack of proper innate sensing inside the tumor microenvironment could reduce both innate and adaptive immunity, which remains a critical cause of immunotherapy failure in various tumor treatments. Double-stranded DNA (dsDNA) has been evidenced to be a promising immunostimulatory agent to induce type I interferons (IFN-Is) production for innate immunity activation through the cyclic GMP-AMP synthase (cGAS)-stimulator of interferon genes (STING) signaling pathway, yet the unsatisfactory delivery and susceptibility to nuclease degradation hindered its feasibility for further clinical applications. Herein, we report on the constructed tumor microenvironment-responsive DNA-based nanomedicine loaded by dendritic mesoporous organosilica nanoparticles (DMONs), which provide efficient delivery of dsDNA to induce intratumoral IFN-Is production for triggering innate sensing for enhanced anti-tumor immunotherapy. Extensive in vitro and in vivo evaluations have demonstrated the dramatic IFN-Is production induced by dsDNA@DMONs in both immune cells and tumor cells, which facilitates dendritic cells (DCs) maturation and T cells activation for eliciting the potent innate immune and adaptive immune responses. Desirable biosafety and marked therapeutic efficacy with a tumor growth inhibition (TGI) of 51.0% on the murine B16-F10 melanoma model were achieved by the single agent dsDNA@DMONs. Moreover, dsDNA@DMONs combined with anti-PD-L1 antibody further enhanced the anti-tumor efficacy and led to almost complete tumor regression. Therefore, this work highlighted the immunostimulatory DNA-based nanomedicine as a promising strategy for overcoming the resistance to immunotherapy, by promoting the IFN-Is production for innate immunity activation and remodeling the tumor microenvironment.

**Graphical abstract:**

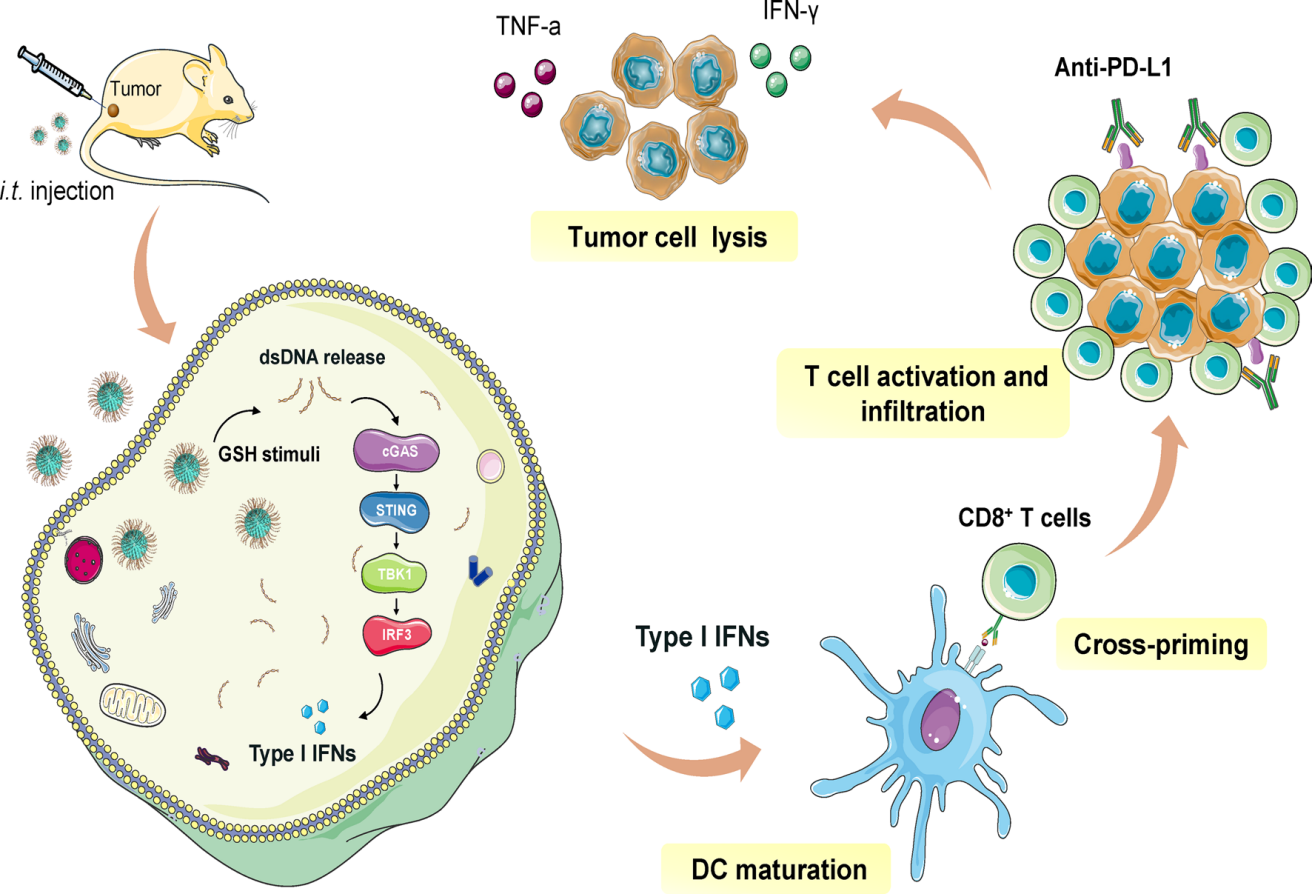

**Supplementary Information:**

The online version contains supplementary material available at 10.1186/s12951-023-02132-6.

## Introduction

Cancer therapy has evolved considerably in recent decades, significantly improving the outcomes and quality of life for patients [1]. In addition to surgery, traditional cancer treatments such as chemotherapies, radiation therapies, and targeted therapies, are suffering from serious challenges such as severe systemic side effects and high treatment costs [2]. Recently, cancer immunotherapies have achieved remarkable strides in the past few years, as evidenced by the success of immune checkpoint blockade (ICB) therapies [3, 4]. However, only a small subset of cancer patients show durable clinical responses to ICB therapies and gain key outcome benefits like progression-free survival (PFS) and overall survival (OS) [5, 6]. Moreover, the magnitude of clinical benefits of ICB is highly variable both across different cancer types and between individual patients [7]. The resistance to ICB is in part due to the underlying immunosuppressive nature of the “cold” tumor, which is characterized by the low infiltration of tumor-infiltrating lymphocytes (TILs), low tumor mutational burden (TMB) and low major histocompatibility complex (MHC) class I expression in the tumor microenvironment (TME) [8, 9].

As an emerging efficient cancer immunotherapeutic modality, immunostimulatory therapies that activate innate sensing pathways are of great promise for overcoming the resistance to tumor immunotherapies by remodeling the TME [10–12]. As the first line of host defense against invading pathogens or dangers, the innate immune response initiation is equipped with pattern-recognition receptors (PRRs) which recognize pathogen-associated molecular patterns (PAMPs) or damage-associated molecular patterns (DMAPs) [13, 14]. The cyclic GMP-AMP synthase (cGAS)-stimulator of interferon genes (STING) signaling pathway, sensing cytoplasmic double-stranded DNA (dsDNA) of both foreign and self-origin, has been established as an essential mechanism implicated in the innate sensing [15–17]. Briefly, cGAS activated by the cytoplasmic dsDNA, catalyzes the synthesis of 2′-3′ cyclic GMP-AMP (cGAMP) and activates the adaptor protein STING, inducing the production of type I interferons (IFN-Is) and secretion of pro-inflammatory cytokine to trigger the innate immune response [18]. Growing evidence has indicated that IFN-Is play a key role in promoting the activation and maturation of dendritic cells (DCs), enhancing the antigen presentation for T cell priming, facilitating tumor immune infiltration, and in turn eliciting the anti-tumor immune responses [19]. Although dsDNA or other cyclic dinucleotides have been evidenced to be efficient immunostimulatory agents for cGAS-STING activation and IFN-Is production for eliciting anti-tumor immune responses, the desirable delivery and exploitation of cyclic dinucleotides remain very challenging due to their negatively charged nature and susceptibility to nuclease degradation [19, 20].

Traditional DNA delivery systems like lipid nanoparticles or cationic polymers often face severe challenges of poor stability and possible fast clearance, which is unfavorable for the effective protection and in vivo delivery of DNA [21, 22]. Dendritic mesoporous silica nanoparticles have motivated extensive research interest as versatile drug delivery systems of a broad range of drugs such as small molecules, proteins and genes, benefiting from their unique central radial pore structure with high surface area for enhanced drug loading efficiency, as well as desirable biocompatibility and feasible surface modification [23, 24]. Especially, dendritic mesoporous organosilica nanoparticles (DMONs) with structurally integrated disulfide bonds, which can be cleaved by glutathione (GSH), can achieve a TME-responsive biodegradation and drug release owing to the high expression level of GSH in cancer cells [25].

**Scheme 1.**
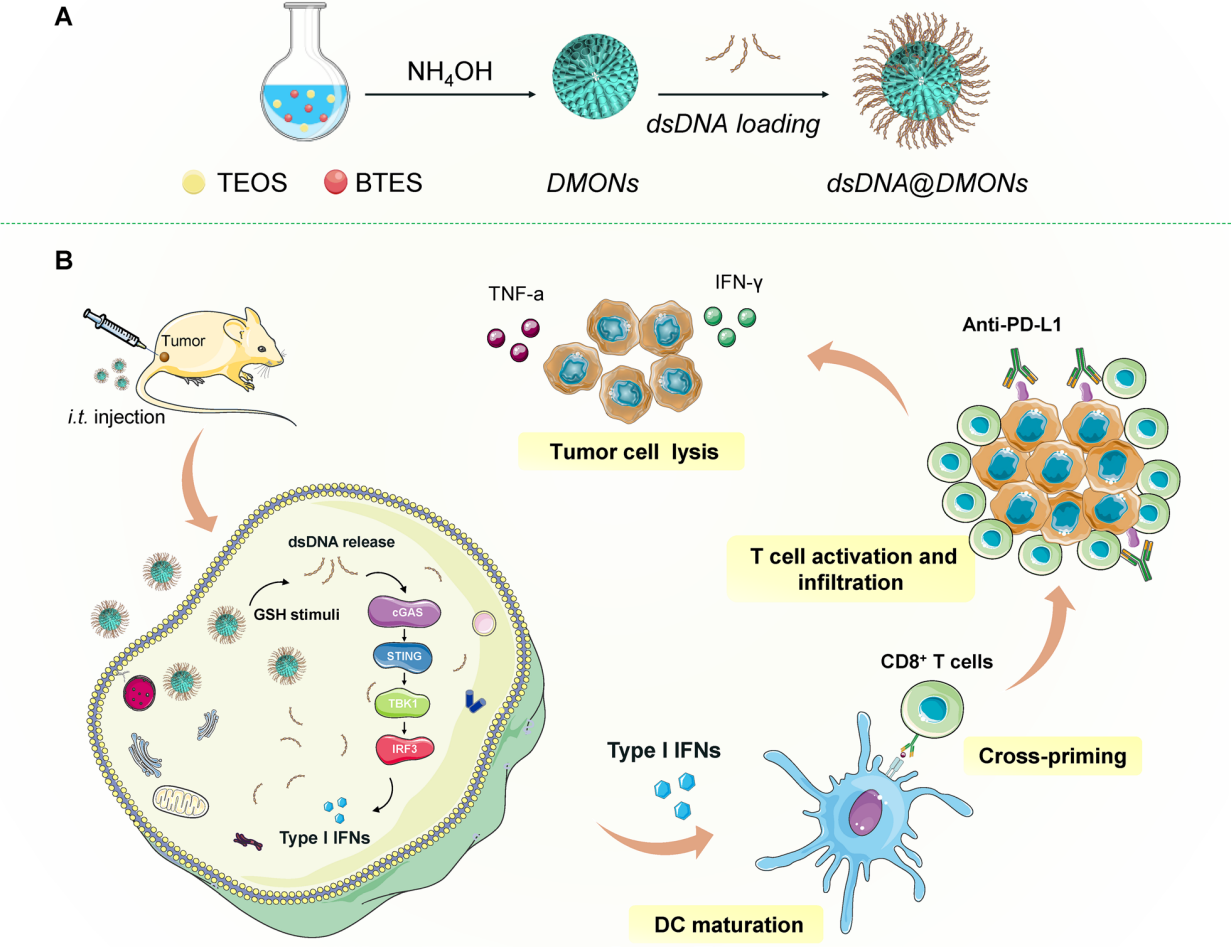
Schematic illustration of DNA-based nanomedicine triggers innate sensing for enhanced immunotherapy. **A** The construction of dsDNA@DMONs nanomedicine. **B** The constructed dsDNA@DMONs induce IFN-Is production and activate innate and adaptive immune responses for anti-tumor immunotherapy

Herein, we report on the rationally designed TME-responsive immunostimulatory nanomedicine dsDNA@DMONs for efficient tumor immunotherapy, based on the DMONs with small particle size and large pore size, which facilitate the efficient intratumoral delivery of dsDNA in a TME-responsive way, for eliciting innate immunity and anti-tumor responses. The dsDNA@DMONs release dsDNA in response to GSH, which triggers innate sensing via the cGAS-STING pathway, inducing the production of IFN-Is to promote DCs maturation, antigen-priming, and T cell activation. As a result, the potent activation of adaptive anti-tumor T cell response can be achieved by the dsDNA@DMONs, and the therapeutic efficacy can be further enhanced in combination with ICB therapy (Scheme 1).

## Results and discussion

### Synthesis and characterization of dsDNA@DMONs nanomedicine

DMONs were synthesized by a triethanolamine (TEA)-catalyzed co-condensation reaction of tetraethyl orthosilicate (TEOS) and bis[3-(triethoxysilyl)propyl] tetrasulfide (BTES) based on the Stöber mechanism and sol–gel chemistry, with sodium salicylate as structural directing agents [26]. The introduction of BTES as an organosilica precursor facilitates the integration of the –S–S–S–S– functional group into the silica skeleton backbone, which in turn improves the biocompatibility and physiological stability of DMONs. Notably, benefiting from the redox reactivity of the disulfide group to GSH, the DMONs were expected to possess specific biodegradability in TME where the GSH is relatively higher than normal tissues [27, 28].

**Fig. 1:**
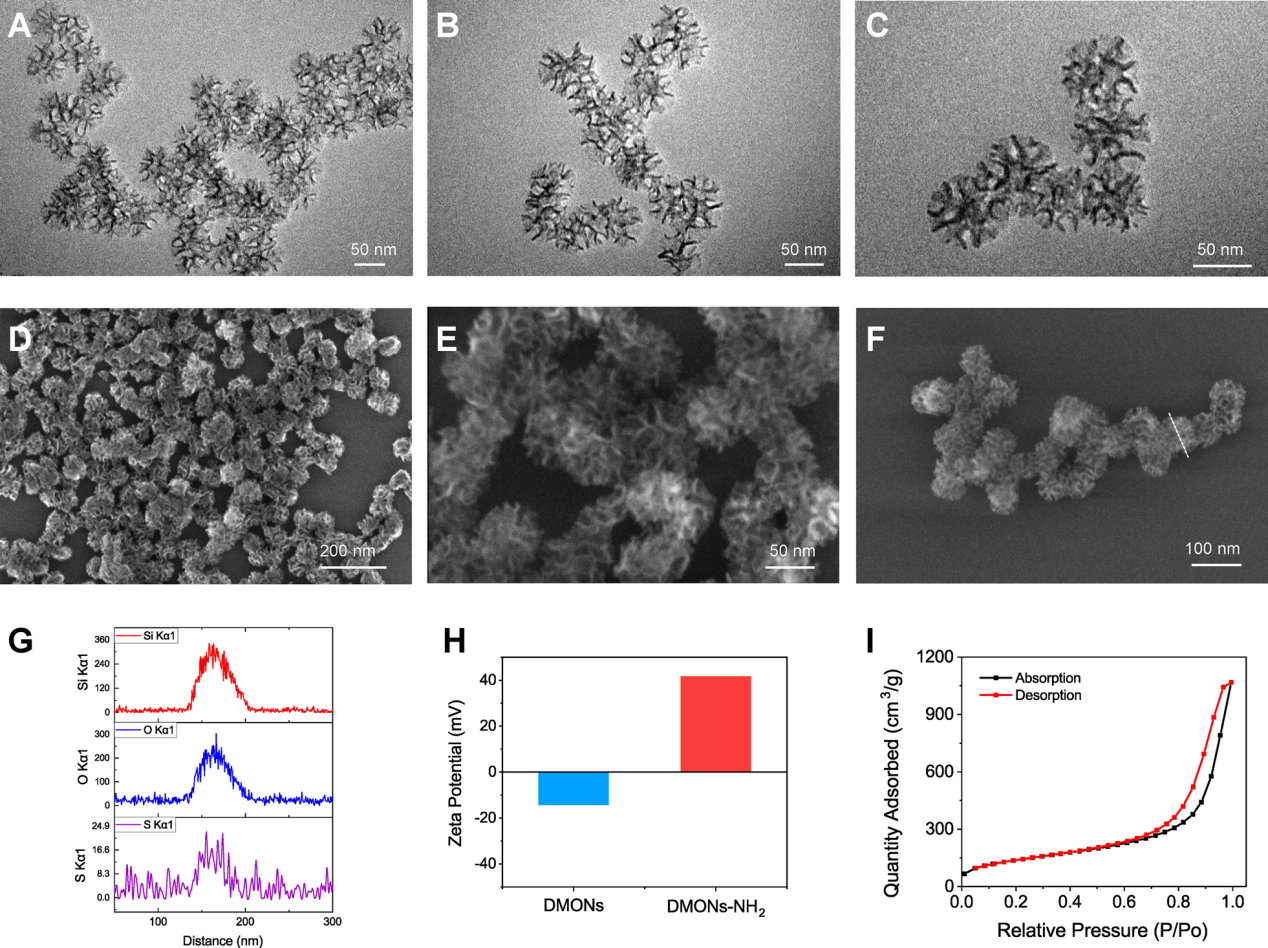
The morphology and structure characterization of DMONs. **A**–**C** TEM images of DMONs in low, medium, and high magnifications. **D**, **E** SEM images of DMONs in low and high magnifications. **F** The SEM images and **G** the corresponding elemental Si, O and S EDS scanning profiles of the DMONs marked with a white dashed line. **H** The Zeta potential of DMONs and DMONs-NH_2_. **I** N_2_ absorption–desorption isotherm of DMONs

Transmission electron microscopy (TEM) images demonstrated the unique dendritic central-radial pore structure of DMONs with a uniform diameter of about 50 nm **(**Fig. 1A–C**)**. Scanning electron microscopy (SEM) images exhibited the distinct wrinkle structure of DMONs **(**Fig. 1D–F**)**, and their corresponding chemical composition was further confirmed by energy dispersive X-ray (EDS) scan profile of Si, O and S elements **(**Fig. 1G), demonstrating the successful integration of organosilica component into the DMONs skeleton. DMONs were modified with anchoring amino groups on the surface to obtain the positively charged DMONs-NH_2_ with a positive Zeta potential of 41.9 mV (Fig. 1H), which facilitates the binding of negatively charged dsDNA through electrostatic interaction. The nitrogen adsorption–desorption isotherms verified the well-defined mesoporous structure of DMONs with a large Brunauer–Emmett–Teller (BET) surface area of 513.3 m^2^/g, while the pore size was calculated to be 12.9 nm, according to the pore-size distribution analysis (Fig. 1I, Additional file 1: Figure S1). The unique branched structure of DMONs with small particle size, high specific surface area, and positively charged surface are highly favorable for the loading and endocytosis of dsDNA while protecting it against degradation by nucleases [29, 30].

**Fig. 2:**
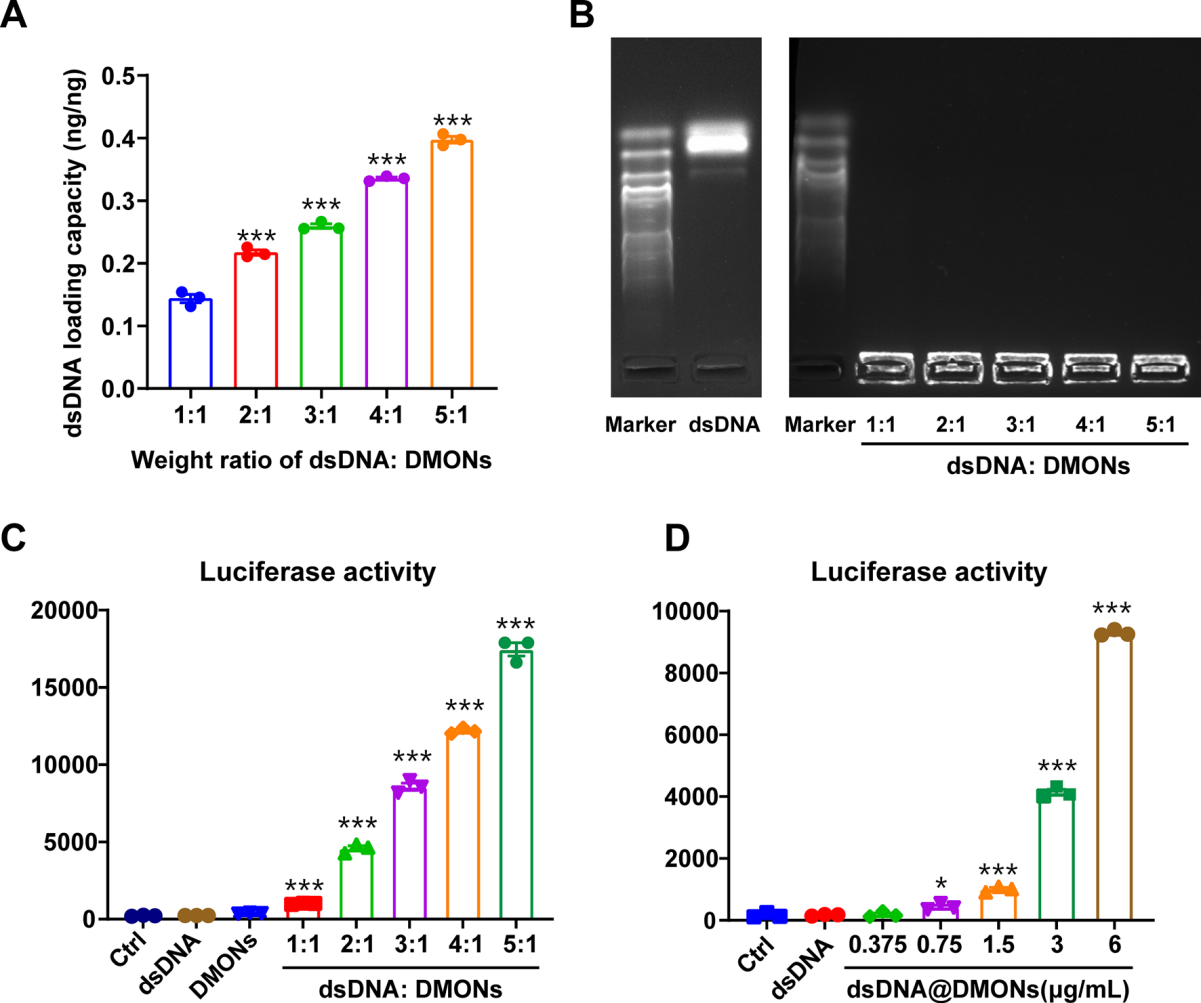
Confirmation of dsDNA binding to DMONs and evaluation of the innate immune activation of dsDNA@DMONs.** A** The dsDNA loading capacity of dsDNA@DMONs at various weight ratios of dsDNA: DMONs with a fixed concentration of DMONs (Si: 100 μg/mL). **B** Images of agarose gel electrophoresis of free dsDNA and dsDNA@DMONs at various weight ratios of dsDNA: DMONs, with Trans2K Plus DNA Marker. **C** The luciferase secretion levels of RAW-Lucia ISG cells treated with dsDNA (2.4 μg/mL), DMONs (Si: 6 μg/mL) and dsDNA@DMONs (Si: 6 μg/mL) with various weight ratios of dsDNA: DMONs of 1:1 to 5:1. **D** The luciferase secretion levels of RAW-Lucia ISG cells treated with dsDNA and dsDNA: DMONs of various concentrations. Data are shown as mean ± SEM (n ≥ 3). P value was calculated by unpaired Student's t-test in **A, C** and **D**. (*p < 0.05; ***p < 0.001)

The dsDNA was loaded into DMONs by electrostatic interaction, and the loading capacity at various weight ratios of dsDNA: DMONs was determined by UV–vis spectra. When the weight ratio of dsDNA to DMONs increased from 1:1 to 5:1 with a fixed concentration of DMONs, the loading capacity increased from 0.15 ng/ng to 0.40 ng/ng accordingly (Fig. 2A). The binding of dsDNA to DMONs was further confirmed by agarose gel electrophoresis. For dsDNA@DMONs with weight ratios of dsDNA: DMONs ranging from 1:1 to 5:1, obvious bands were observed to be dwelled in the sample wells (Fig. 2B), indicating the robust binding of dsDNA to DMONs which hindered the migration of dsDNA. Subsequently, the innate immunostimulatory activity of the dsDNA@DMONs at various weight ratios was evaluated based on the RAW-Lucia ISG reporter cell system. After being treated with dsDNA@DMONs for 24 h, the level of IRF-induced luciferase in the cell culture supernatant was monitored by a microplate reader. The results showed that the luciferase activity of reporter cells was significantly enhanced, indicating the dramatic IFN-Is production induced by dsDNA@DMONs (Fig. 2C). Moreover, the IFN-Is production activity showed an upward trend with the increase of the weight ratios of dsDNA: DMONs from 1:1 to 5:1, which is consistent with the increasing dsDNA loading capacity. Thereafter, the loading weight ratio of dsDNA: DMONs was determined to be 5:1, considering the optimal loading capacity and immunostimulatory activity. Importantly, the reporter cells exhibited increasing luciferase activity after being treated with dsDNA@DMONs of increasing concentrations (Fig. 2D), indicating that the dsDNA@DMONs can induce the IFN-Is production in a dose-dependent manner. The in vitro dsDNA release profile was investigated in simulated body fluid (SBF) with various GSH concentrations. It was observed that the release rate was markedly boosted in the presence of GSH (10 mM) and almost complete dsDNA was released from the DMONs within 96 h, indicating the GSH-triggered degradability and drug release of DMONs benefiting from the redox reactivity of the disulfide group within the structure (Additional file 1: Figure S2).

The time-coursed cell endocytosis of DMONs was evaluated by confocal laser confocal microscopy (CLSM). In both the B16-F10 tumor cell line and DC2.4 dendritic cell line, the red fluorescence could be observed within 30 min incubation, and increased gradually in 6 h, indicating the fast internalization of DMONs in both tumor cells and immune cells (Additional file 1: Figure S3). Furtherly, the cytotoxicity of DMONs was evaluated by a standard CCK-8 method. After treatments of DMONs with a maximum concentration of 6 μg/mL, no significant decrease in RAW 264.7 cell viability was observed compared to the control group (Additional file 1: Figure S4). The above results indicated that the constructed dsDNA@DMONs with high dsDNA loading capability, feasible cell endocytosis and desirable biocompatibility, could efficiently induce the IFN-Is production of RAW 264.7 reporter cells.

### The dsDNA@DMONs trigger the activation of type I interferon signaling in DC cells

**Fig. 3:**
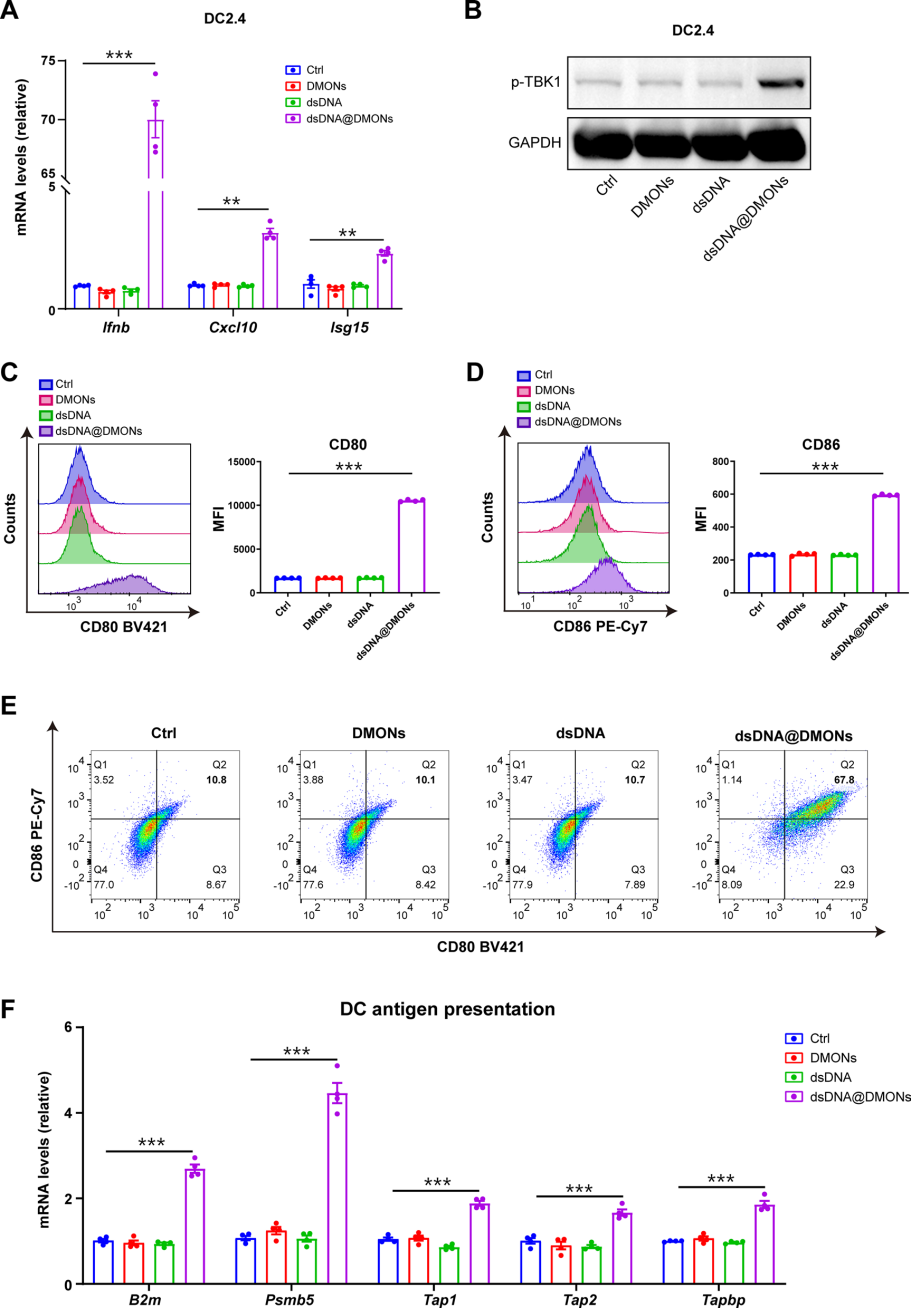
The dsDNA@DMONs trigger the activation of type I interferon signaling and promote the maturation and antigen presentation ability of DC cells. **A** RT-qPCR analysis of the gene expression of *Ifnb*, *Cxcl10*, and *Isg15* in DC2.4 cells treated with PBS (Control, Ctrl), DMONs (Si: 6 μg/mL), dsDNA (2.4 μg/mL) or dsDNA@DMONs (Si: 6 μg/mL) for 24 h. **B **Western blot of p-TBK1 in DC2.4 cells treated with PBS, DMONs (Si: 6 μg/mL), dsDNA (2.4 μg/mL) or dsDNA@DMONs (Si: 6 μg/mL) for 24 h. GAPDH serves as a loading control. **C–E** Mean fluorescent intensity (MFI) of **C** CD80 and **D** CD86 by FACS analysis in DC2.4 cells treated with PBS, DMONs (Si: 6 μg/mL), dsDNA (2.4 μg/mL) or dsDNA@DMONs (Si: 6 μg/mL) for 24 h, scatter plots of CD80 and CD86 were shown in **E**. **F** RT-qPCR analysis of the gene expression of *B2m*, *Psmb5*, *Tap1*, *Tap2*, and *Tapbp* in DC2.4 cells treated with PBS, DMONs (Si: 6 μg/mL), dsDNA (2.4 μg/mL) or dsDNA@DMONs (Si: 6 μg/mL) for 24 h. Data are shown as mean ± SEM (n ≥ 3). P value was calculated by unpaired Student's t-test in **A, C, D** and** F**. (**p < 0.01; ***p < 0.001)

To further investigate the IFN-Is signal pathway activation by dsDNA@DMONs, we used RT-PCR to determine the expression level of genes involved in the IFN-Is signaling pathway such as *Ifnb*, *Cxcl10* and *Isg15* in DC2.4 cells. The results showed that the expression of *Ifnb*, as well as the transcription level of the downstream genes *Cxcl10* and *Isg15,* were upregulated by dsDNA@DMONs, while the DMONs or dsDNA did not cause any significant change in IFN-Is expression compared to the control group (Fig. 3A). Meanwhile, the western blotting results showed that the p-TBK1 protein expression level, which is an important modulator for the IFN-Is production, has been significantly enhanced by dsDNA@DMONs treatment compared to the control group (Fig. 3B). The activation of IFN-Is signaling pathway will promote the maturation and antigen presentation functionality of DCs [31]. Flow cytometry was introduced to explore the expression of surface biomarkers CD86 and CD80. Significant upregulation of CD86 and CD80 molecules of DC2.4 cells was observed after dsDNA@DMONs treatment compared to the control group (Fig. 3C–E). However, the expression levels of CD80 and CD86 in DC2.4 cells exhibited negligible change after being treated with DMONs or dsDNA. As a kind of professional antigen-presenting cells, the antigen-presentation ability of DC cells was related to the maturation status. The expression of genes involved in antigen presentation was examined by RT-qPCR, which showed that the dsDNA@DMONs treatment elicited the instinct upregulation of the *B2m*, *Psmb5*, *Tap1*, *Tap2*, and *Tapbp* genes of DC2.4 cells compared to the control group (Fig. 3F).

### The dsDNA@DMONs induced the activation of type I interferon signaling and enhanced the antigen presentation ability of tumor cells

**Fig. 4:**
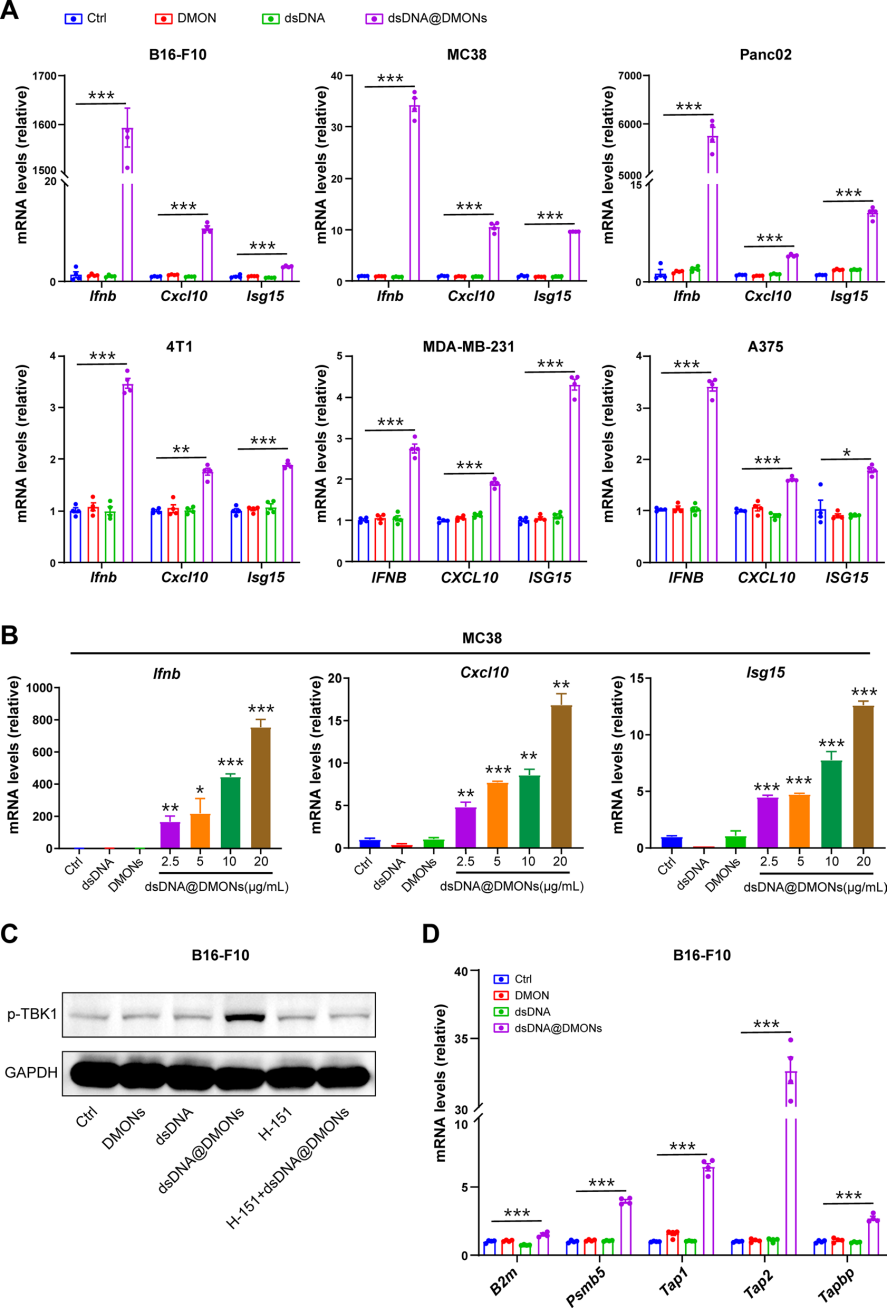
The dsDNA@DMONs induced the activation of type I interferon signaling and enhanced the antigen presentation ability of tumor cells. **A** RT-qPCR analysis of the gene expression of *Ifnb*, *Cxcl10*, and *Isg15* in marine cell lines MC38, B16-F10, 4T1, Panc02 treated with PBS, DMONs (Si: 6 μg/mL), dsDNA (2.4 μg/mL) or dsDNA@DMONs (Si: 6 μg/mL) for 24 h; and *IFNB*, *CXCL10*, and *ISG15* in human cell lines MDA-MB-231 and A375 treated with PBS, DMONs (Si: 6 μg/mL), dsDNA (2.4 μg/mL) or dsDNA@DMONs (Si: 6 μg/mL) for 24 h. **B** RT-qPCR analysis of the gene expression of *Ifnb*, *Cxcl10*, and *Isg15* in B16-F10 treated with the indicated concentration of dsDNA@DMONs for 24 h. **C** Western blot of p-TBK1 in B16-F10 cells treated with PBS, DMONs (Si: 6 μg/mL), dsDNA (2.4 μg/mL) or dsDNA@DMONs (Si: 6 μg/mL) with or without H-151 (2 μM) for 24 h. GAPDH serves as a loading control. **D** RT-qPCR analysis of the gene expression of *B2m*, *Psmb5*, *Tap1*, *Tap2*, and *Tapbp* in B16-F10 cells treated with PBS, DMONs (Si: 6 μg/mL), dsDNA  (2.4 μg/mL) or dsDNA@DMONs (Si: 6 μg/mL) for 24 h. Data are shown as mean ± SEM (n ≥ 3). P value was calculated by unpaired Student's t-test in **A**,** B** and** D**. (*p < 0.05; **p < 0.01; ***p < 0.001)

As tumor cell-derived IFN-Is production plays a key role in anti-tumor immune response [19, 32], the capability to induce IFN-Is production by dsDNA@DMONs was evaluated in multiple mouse cancer cell lines, including B16-F10 melanoma cells, MC38 colorectal cancer cells, 4T1 breast cancer cells, and Panc02 pancreatic cancer cells. The results showed that dsDNA@DMONs treatment could upregulate the expression of *Ifnb* and increase the transcription of downstream genes *Cxcl10* and *Isg15* compared to the control (Fig. 4A), while DMONs or dsDNA only could not efficiently stimulate the production of *Ifnb*, *Cxcl10* and *Isg15 genes*. For human breast cancer cell line MDA-MB-231 and melanoma cell line A375, the treatment of dsDNA@DMONs could also arouse the upregulation of *IFNB*, *CXCL10* and *ISG15* genes. The above results proved that dsDNA@DMONs possess the ability to activate the IFN-Is signal pathway in multiple tumor cells of both mice and humans. Importantly, the IFN-Is production in tumor cells was found to be induced by dsDNA@DMONs in a dose-dependent manner, indicating the key role of the immunostimulatory dsDNA@DMONs (Fig. 4B). As the key downstream protein in the cGAS-STING pathway, p-TBK1 expression was further examined by western blotting on B16-F10 cells after various treatments. The results showed that DMONs or dsDNA only was invalid to facilitate the phosphorylation of TBK1 in comparison with the control group, while significant upregulation of p-TBK1 was observed after dsDNA@DMONs treatments (Fig. 4C). It is worth noting that the induction of phosphorylation of TBK1 by dsDNA@DMONs could be markedly inhibited by STING inhibitor H-151, which indicates that dsDNA@DMONs promote p-TBK1 expression by activating the cGAS-STING pathway. Taken together, these results demonstrated that dsDNA@DMONs could effectively induce the production of IFN-Is by initiating cGAS-STING sensing signaling. Considering the IFN-Is could regulate the immune escape of tumor cells by affecting their antigen priming [33, 34], the expression levels of tumor antigen presentation-related genes were examined, showing that the dsDNA@DMONs treatment could significantly induce the expression of genes *B2m*, *Psmb5*, *Tap1*, *Tap2* and *Tapbp* compared with the control group (Fig. 4D). The above results demonstrated that dsDNA@DMONs could activate the IFN-Is signal pathway and enhance the antigen presentation ability in tumor cells.

### In vivo therapeutic effect of dsDNA@DMONs immunostimulatory nanomedicine

**Fig. 5:**
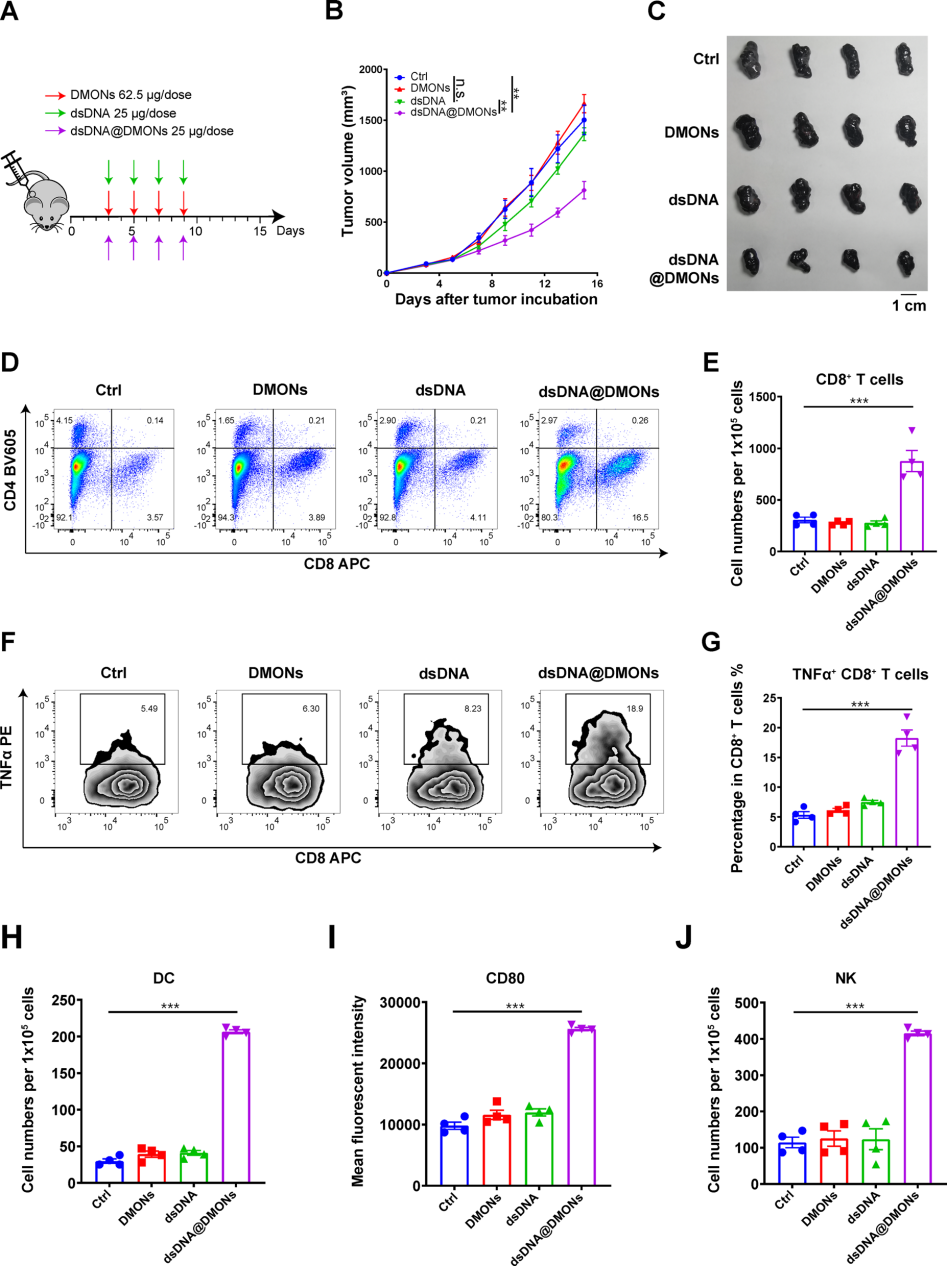
The dsDNA@DMONs exhibit potent anti-tumor efficacy and remodel the tumor immune microenvironment.** A** Schematic illustration of the dosing regimen, a subcutaneous tumor model was established on C57BL/6 J mice using B16-F10 cells, and different treatments were given to each group (n = 6) after tumor formation. **B** Growth curve of the tumor volume of mice in each group. **C** Representative photographs of B16-F10 tumor tissues dissected from each group after the therapeutic period. **D** Flow cytometry analysis showing the percentage of infiltrating CD8^+^ and CD4^+^ T cells in CD45^+^ cells in each group. **E** Number of CD8^+^ T lymphocytes per 100,000 cells in tumor tissues from each group analyzed by flow cytometry. **F** Flow cytometric analysis of TNFα^+^CD8^+^ lymphocytes out of the total CD8^+^ T lymphocytes and **G** the respective statistical analysis. **H** Analysis of the number of DCs per 100,000 cells in tumor tissues using flow cytometry. **I** Expression of CD80 on the surface of DCs. **J** Number of natural killer (NK) cells. Data are shown as mean ± SEM (n ≥ 3). P value was calculated by unpaired Student's t-test in **E, G**-**J,** or two-way ANOVA in** B**. (n.s., not significant, p > 0.05; **p < 0.01; ***p < 0.001).

Encouraged by the in vitro experimental results, which have evidenced the potential of dsDNA@DMONs serving as an ideal immunostimulatory nanomedicine for efficiently inducing innate sensing activation both in immune cells and tumor cells, we further verified the in vivo anti-tumor efficacy of dsDNA@DMONs on subcutaneously established murine B16-F10 melanoma xenograft model. Mice were randomly divided into four groups three days after tumor inoculation and the tumor volume of each single mouse was monitored using a digital caliper. When the tumor volumes reached about 60 mm^3^, different administrations of PBS (control), DMONs (Si: 62.5 μg/dose), dsDNA (25 μg/dose) and dsDNA@DMONs (Si: 62.5 μg/dose, dsDNA: 25 μg/dose) were intratumorally administrated respectively to each group on every other day, for a total of 4 doses (Fig. 5A). Compared with the control group, DMONs or free dsDNA didn’t show any therapeutic effect on tumor suppression, while dsDNA@DMONs treatment has significantly delayed tumor growth with a tumor growth inhibition (TGI) of 51.0% (Fig. 5B, C, Additional file 1: Figure S5). The flow cytometric analysis of Ki-67 antibody staining in tumor cells revealed the suppressed proliferative activity of tumor cells by dsDNA@DMONs, while there were minimal significant effects on cell proliferation in the control group (Additional file 1: Figure S6). The body weights of mice in four groups were monitored during the therapeutic period, and dsDNA@DMONs treatment did not cause a significant decrease in mice body weights, indicating the high biocompatibility of dsDNA@DMONs (Additional file 1: Figure S7). The mice were euthanized at the end of the experiment, and hematoxylin and eosin (H&E) staining of the major organs (heart, liver, spleen, lung, and kidney) showed that the treatments of DMON, dsDNA, and dsDNA@DMONs didn’t cause any noticeable pathological side effects on major organs of mice, compared to the control group (Additional file 1: Figure S8).

The immunostimulatory nanomedicine dsDNA@DMONs are designed to induce the production of IFN-Is, which further remodels the tumor immune microenvironment by modulating the functions of varieties of immune cells. The immune microenvironment was further analyzed by flow cytometric analysis of tumor tissues to verify the immunostimulatory activity of dsDNA@DMONs. A significantly increased percentage of cytotoxic T lymphocytes (CD8^+^ T cells) was observed with dsDNA@DMONs treatment (49.4%), compared to control (29.6%), DMONs (30.9%), and free dsDNA (33.5%) group (Fig. 5D, E). The TNFα expression level was further analyzed to clarify the cytotoxicity of CD8^+^ T cells, which indicated that the dsDNA@DMONs could enhance the percentage of TNFα^+^CD8^+^ T cells, while DMONs or free dsDNA caused a minimal increase in the TNFα^+^CD8^+^ T cells percentage compared to the control group (Fig. 5F, G). The above results showed that the dsDNA@DMONs could efficiently increase the infiltration and activation of CD8^+^ T lymphocytes. Moreover, the dsDNA@DMONs treatment could promote the tumor DCs infiltration and maturation as indicated by the CD80 expression level on DC cells (Figs. 5H, I), as well as the infiltration of NK cells regulated by the IFN-Is signaling activation (Fig. 5J). The above analysis results show that dsDNA@DMONs could significantly remodel the tumor immune microenvironment, promoting the infiltration and activation of multiple immunostimulatory immune cells like CD8^+^ T lymphocytes, DC cells and NK cells. This potent immunostimulatory activity of dsDNA@DMONs may be attributed to the effective protection and intratumoral delivery of dsDNA by DMONs, with the TME-responsive dsDNA release locally in the tumor region, which elicits the potent tumor-specific innate sensing and adaptive immune responses and results in the superior tumor inhibition effects with desirable therapeutic biosafety.

**Fig. 6:**
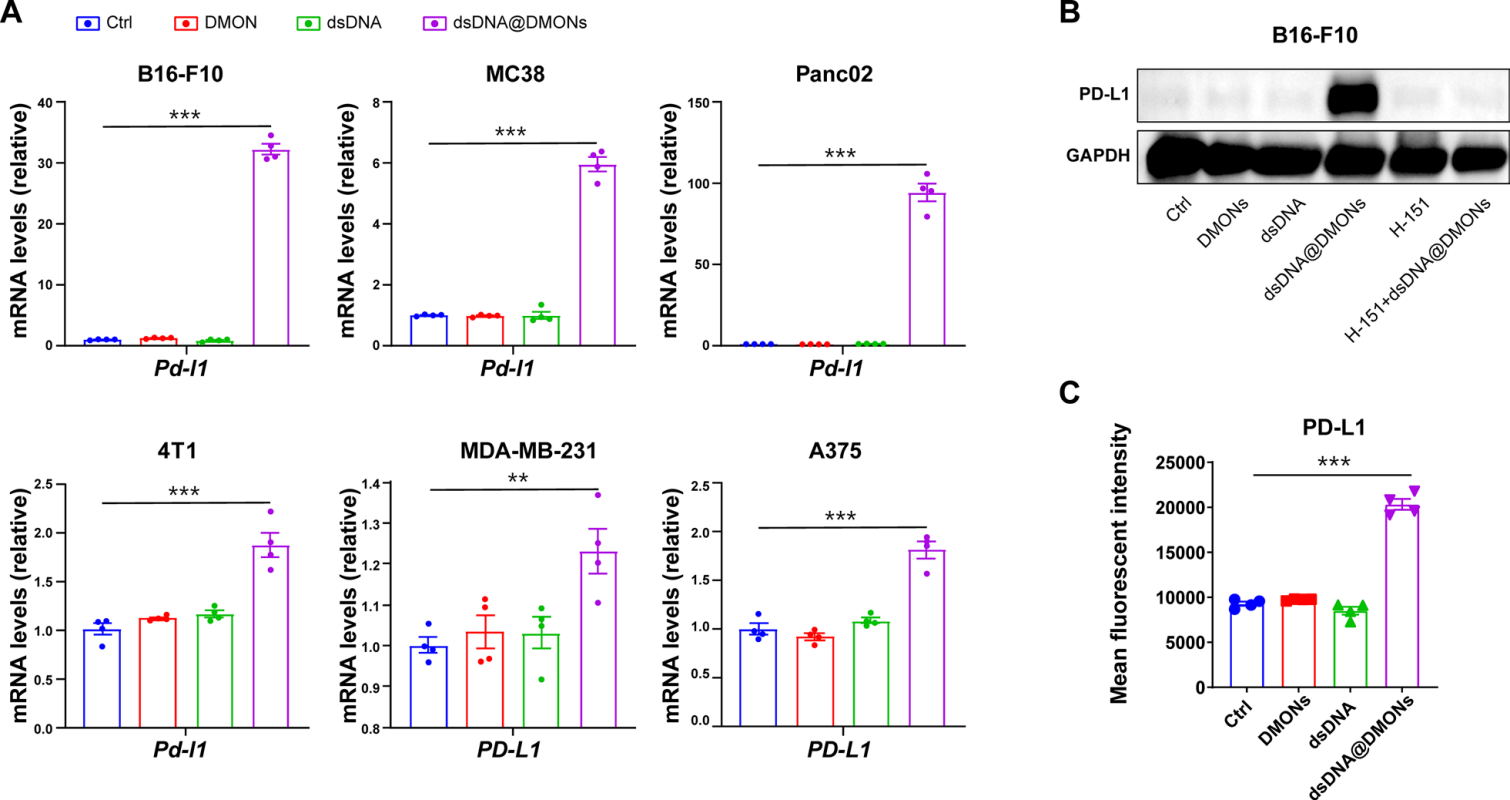
The dsDNA@DMONs induced PD-L1 expression in tumor cells. **A** RT-qPCR analysis of the gene expression of *Pd-l1* in MC38, B16-F10, 4T1, Panc02, MDA-MB-231 and A375 cells treated with PBS, DMONs (Si: 6 μg/mL), dsDNA (2.4 μg/mL) or dsDNA@DMONs (Si: 6 μg/mL) for 24 h. **B** Western blot assays of PD-L1 in B16-F10 cells treated with PBS, DMONs (Si: 6 μg/mL), dsDNA (2.4 μg/mL) or dsDNA@DMONs (Si: 6 μg/mL) with or without H-151 (2 μM) for 24 h. GAPDH serves as a loading control. **C** Analysis of PD-L1 expression on tumor cells in tumor tissues using flow cytometry. Data are shown as mean ± SEM (n ≥ 3). P value was calculated by unpaired Student's t-test in **A** and** C**. (**p < 0.01; ***p < 0.001)

As inspired by the previous studies which reported that the activation of IFN-Is signal pathway could upregulate PD-L1 expression through IFNAR1-STAT1 signaling [35], the PD-L1 expression in tumor cells was examined to reveal the effect of dsDNA@DMONs treatments. The in vitro experiments showed that the *Pd-l1* gene expression level could be significantly upregulated by dsDNA@DMONs, rather than DMONs or dsDNA, as verified on multiple murine cell lines like MC38, B16-F10, 4T1, Panc02, as well as human cell lines like MDA-MB-231, and A375 cells (Fig. 6A). Moreover, the western blotting and flow cytometry analysis were conducted to evaluate the PD-L1 expression in tumor tissues after in vivo experiments of various treatments. In accordance with the in vitro study, the dsDNA@DMONs treatment could enhance the PD-L1 protein expression compared to controls by activating the cGAS-STING pathway (Fig. 6B, C). Notably, the high PD-L1 expression level was found to be correlated with better responses to anti-PD-1/PD-L1 immunotherapy, as evidenced in extensive clinical studies [36]. The upregulation effects of dsDNA@DMONs on PD-L1 expression suggested that the combination of the immune checkpoint inhibitors with dsDNA@DMONs may be an effective strategy for achieving favorable anti-tumor responses.

### Enhanced anti-tumor therapy by immunostimulatory dsDNA@DMONs combined with anti-PD-L1 blockade

**Fig. 7:**
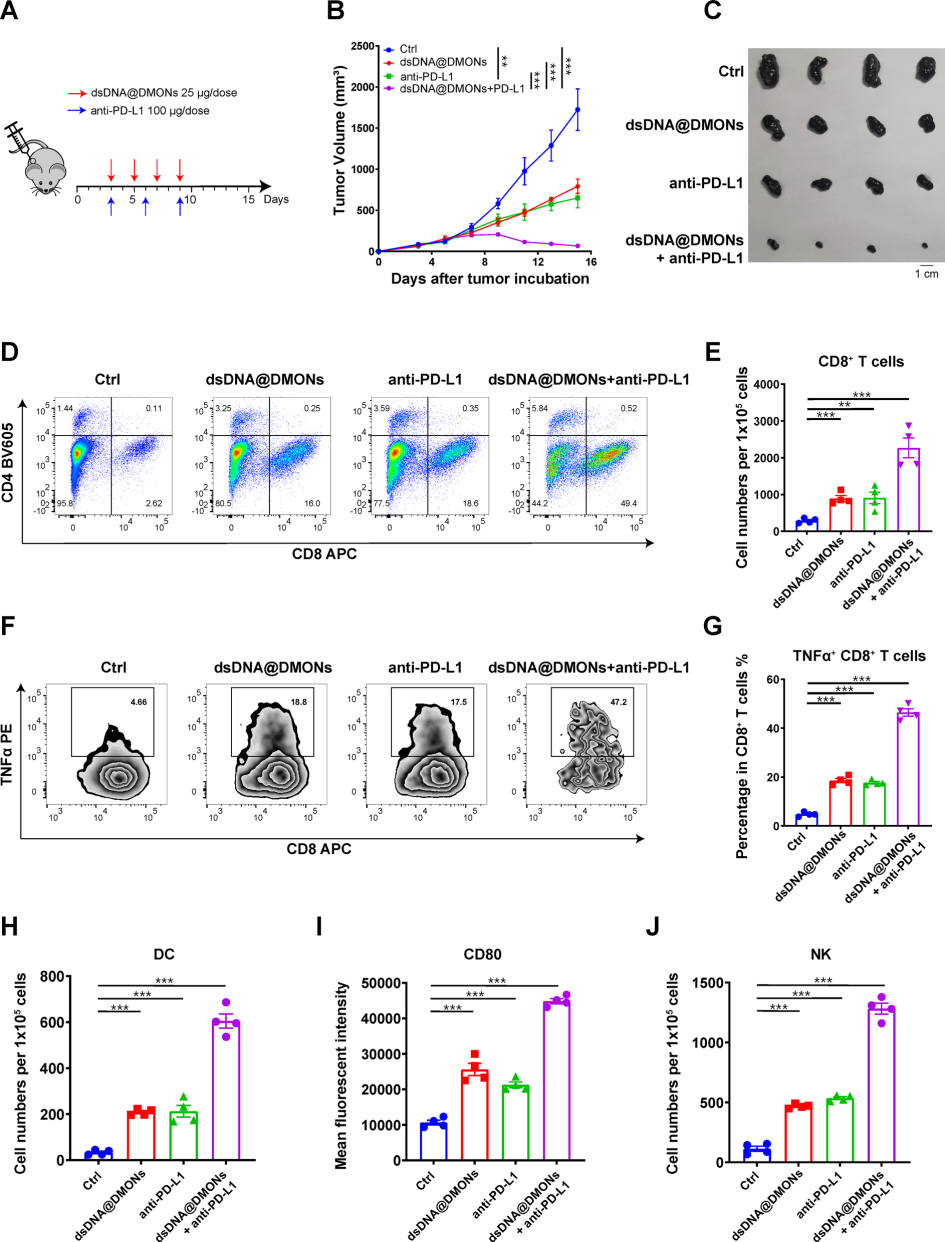
Enhanced anti-tumor therapy by immunostimulatory dsDNA@DMONs combined with anti-PD-L1 blockade.** A** Schematic illustration of the dosing regimen, a subcutaneous tumor model was established on C57BL/6J mice using B16-F10, and after tumor formation, each group (n ≥ 5) was given different treatments according to the illustrated regimen. **B** Tumor growth curve of mice in each group. **C** Representative photographs of B16-F10 tumor tissues dissected from each group. **D** Flow cytometry analysis showing the percentage of CD8^+^ and CD4^+^ T cells in CD45^+^CD3^+^ cells. **E** Number of CD8^+^ T lymphocytes per 100,000 cells in tumor tissues from each group. **F** Flow cytometric analysis of TNFα^+^ CD8^+^ T lymphocytes out of the total CD8^+^ T lymphocytes and **G** the respective statistical analysis. **H** Number of DC cells per 100,000 cells in tumor tissues from each group. **I** Expression of CD80 on the surface of DC cells. **J** Number of NK cells per 100,000 cells in tumor tissues from each group. Data are shown as mean ± SEM (n ≥ 3). P value was calculated by unpaired Student's t-test in **E**,** G**–**J** or two-way ANOVA in** B**. (**p < 0.01; ***p < 0.001)

The combination potential of dsDNA@DMONs with the immune checkpoint inhibitor was verified on the subcutaneous melanoma B16-F10 model. After tumor formation, dsDNA@DMONs (Si: 62.5 μg/dose, dsDNA: 25 μg/dose) was intratumorally administrated every other day for a total of four doses, and anti-PD-L1 antibody (100 μg/dose) was injected intraperitoneally every 3 days for a total of three doses (Fig. 7A). The tumor growth curve showed that the dsDNA@DMONs and anti-PD-L1 antibody alone could moderately inhibit tumor growth with the TGI of 55.3% and 60.9%, respectively. While the combination administration of dsDNA@DMONs and anti-PD-L1 caused almost complete regression of tumor volume, yielding a TGI as high as 96.7% (Fig. 7B, C, Additional file 1: Figure S9). And the combination of dsDNA@DMONs and anti-PD-L1 showed superior activity in inhibiting tumor cell proliferation by Ki-67 antibody staining, compared with the single agent of either dsDNA@DMONs of anti-PD-L1 antibody (Additional file 1: Figure S10), with the satisfactory biosafety evidenced by the mice body weights monitoring and H&E staining of the major organs (Additional file 1: Figures S11, S12).

The flow cytometric analysis showed that the combination of dsDNA@DMONs and anti-PD-L1 could encouragingly facilitate the CD8^+^ T cell infiltration (Fig. 7D, E) and activation (Fig. 7F, G). In addition, DCs maturation and NK cells activation were promoted by the combination of dsDNA@DMONs and anti-PD-L1, superior to the effects caused by the single agent (Fig. 7H-J). The above results demonstrated the potent anti-tumor activity of combination therapy of dsDNA@DMONs with PD-L1 antibody, indicating the immunostimulatory nanomedicine dsDNA@DMONs as a promising strategy for overcoming the resistance of immune checkpoint inhibitor therapy.

## Conclusion

Current cancer immunotherapies have been plagued by low responses due to the underlying immunosuppressive TME with limited antigen-priming and low infiltration of lymphocytes. Immunostimulatory therapies provide effective solutions to overcome the resistance to tumor immunotherapies by activating innate sensing pathways and remodeling the TME.

In this work, we report on the construction of TME-responsive immunostimulatory nanomedicine based on dsDNA loaded by biocompatible DMONs with small particle size and large pore size, which provide efficient protection and delivery of dsDNA for triggering intratumoral IFN-Is production to activate innate sensing, enabling the enhanced anti-tumor immunotherapy with marked therapeutic efficacy and biosafety. Comprehensive in vitro and in vivo experimental results showed that the effective intratumoral delivery of dsDNA by DMONs can induce the IFN-Is production in both immune cells and tumor cells, promoting DCs maturation and T cells activation for eliciting the potent innate sensing and adaptive immune responses. Satisfactory tumor inhibition effect on murine B16-F10 melanoma model with desirable therapeutic biosafety was achieved by intratumoral administration of the dsDNA@DMONs. Moreover, the dsDNA@DMONs in combination with anti-PD-L1 antibodies can further enhance the anti-tumor efficacy leading to almost complete tumor regression.

In summary, this study demonstrated that the DNA-based nanomedicine for triggering innate sensing and adaptive immune responses not only establishes a paradigm of immunostimulatory therapy but also takes an important step forward in developing immunostimulatory nanomedicine with TME remodeling activities for enhancing the efficacy and overcoming the resistance of immunotherapy.

## Methods

### Materials and reagents

The reagents used included QUANTI-Luc™ (InvivoGen), DAPI-containing anti-fade medium (P36962, Thermo Fisher Scientific), Gelstain (GS101, Transgen), Trans2K Plus DNA Marker (BM111, Transgen), Direct-zol RNA Miniprep Plus Kit (R2070, ZYMO RESEARCH), PrimeScriptTM RT reagent Kit with gDNA Eraser (RR047A, Takara), DreamTaq polymerase (EP0701, Thermo Scientific), GeneJET PCR purification kit (K0701, Thermo Scientific), 50 × Tris–acetate-EDTA (TAE, ST716, Beyotime Biotechnology), Radio Immunoprecipitation Assay buffer (RIPA, PC101, Epizyme), phosphatase (GRF102; Epizyme), protease inhibitors (GRF101, Epizyme), TRIzol Reagent (15,596,026, Invitrogen). PierceTM BCA Protein Assay Kit (23,225, Thermo Scientific), SDS-PAGE Sample Buffer (P0015F, Beyotime Biotechnology), Multicolor Prestained Protein Ladder (WJ103, Epizyme), BD Pharmingen™ Leukocyte Activation Cocktail (550,583, BD Pharmingen), Fixation/Permeabilization Solution Kit with BD GolgiPlug (555,028, BD Pharmingen), Foxp3/Transcription Factor Staining Buffer Set (00–5523-00, Thermo Scientific), Collagenase I (C10130, Sigma), Hyaluronidase (H3506, Sigma), 25 μg/mL DNase I (DN25-100, Sigma), Triethanolamine (TEA, V900257, Sigma), Hexadecyl trimethyl ammonium bromide(CTAB, H5882, Sigma), Sodium salicylate (NaSal, 54–21-7, Sigma), Tetraethyl orthosilicate (TEOS, 78–10-4, Sigma), Bis[3-(triethoxysilyl)propyl] tetrasulfide (BTES, 40,372–72-3, Sigma-Aldrich), 3-Aminopropyl triethoxysilane (APTES, 919–30-2, Sigma-Aldrich), sulfo-Cyanine5 NHS ester (43,320, Lumiprobe), DreamTaq DNA Polymerase (EP0705, Thermo Scientific), GeneJET PCR Purification Kit (K0701, Thermo Scientific), H-151 (HY-112693, MedChemExpress), Lipofectamine 2000 Transfection Reagent (11,668,019, Thermo Scientific), Simulated body fluid (SBF, R24165, Shanghai yuanye Bio-Technology).

### Antibodies

Anti-PD-L1 (ab213480, Abcam), anti-GAPDH antibody (2118, CST), anti-Phospho-TBK1/NAK antibody (5483, CST), HRP-conjugated secondary antibodies (7074, CST), anti-PD-L1(BE0101, BioXcell), anti-mouse CD16/32 antibody (553,141, BD Pharmingen), Fixable Viability Stain (565,388, BD Pharmingen), anti-CD80-BV421(562,611, BD Pharmingen), anti-CD86-PE-Cy7(560,582, BD Pharmingen), anti-mouse PD-1-PE (135,206, BioLegend), anti-mouse LAG3-PE-Cy7 (125,226, BioLegend), anti-mouse TIM3-BV421 (119,723, BioLegend), anti-mouse FOXP3-BV421 (126,419, BioLegend), anti-mouse Ki67-PE-CY7 (652,426, BioLegend), anti-mouse PD-L1-PE (124,308, BioLegend), anti-mouse CD11b-FITC (101,206, BioLegend), anti-mouse Ly6G-PE (551,461, BD Pharmingen), anti-mouse Ly6C-PE-Cy7 (128,018, BioLegend), anti-mouse F4/80-BV421 (123,132, BioLegend), anti-mouse CD206-APC (141,708, BioLegend), anti-mouse TNFα-PE (554,419, BD Pharmingen), anti-mouse CD3-BV605 (100,237, BioLegend), anti-mouse CD11C-APC (117,310, BioLegend), anti-mouse MHC-II-BV421 (107,632, BioLegend), anti-mouse NK1.1-PE-Cy7 (552,878, BD Pharmingen), anti-mouse CD80-PE (552,769, BD Pharmingen).

### Cells

MC38, B16-F10, 4T1, MDA-MB-231, and A375 cells were acquired from ATCC. Panc02 cells were obtained from the Cell Bank of the Shanghai Institutes for Biological Sciences, Chinese Academy of Sciences (SIBS, CAS). RAW-Lucia ISG cells with an interferon regulatory factor (IRF)-inducible Lucia luciferase reporter construct were acquired from Invivogen. Mouse dendritic cells 2.4 (DC2.4) were kindly provided by Dr. Jing Qian from Jiangsu Academy of Agricultural Sciences (JAAS). All cell lines were routinely tested using a mycoplasma contamination kit (R&D) and cultured in the indicated medium following the manufacturer’s instruction and supplemented with 10% heat-inactivated fetal bovine serum, 100 U/ml penicillin, and 100 U/ml streptomycin. All cells were kept at 37 °C with 5% CO_2_.

### Mice

Female C57BL/6 mice, six weeks old, were obtained from the Shanghai Lingchang Biotechnology Co., LTD. Mice were housed in an SPF animal facility in temperature-controlled rooms at 21 °C, with 45–65% relative humidity and 12-h light/dark cycles at the Shanghai Jiao Tong University School of Medicine. The animal experiments were performed using protocols approved by the Institutional Animal Care and Use Committee.

### Synthesis of DMONs

0.068 g of TEA was added to 25 mL of water and stirred at 300 rpm for 0.5 h at 80 °C, after which 380 mg of CTAB and 168 mg of NaSal were added and stirred for another 1 h. 2 mL of TEOS and 2 mL of BTES were added and continued to stir at 80 °C for 2 h. The products were collected by centrifugation at 20,000 rpm at the end of the reaction, after which the residual reactants were removed by washing three times using water and ethanol. The DMONs were extracted with a mixture solution of HCl and anhydrous ethanol (V _HCl_: V _ethanol_ = 1: 9) at 80 °C for 12 h three times to remove the CTAB. The obtained DMONs were dried under vacuum at room temperature. 100 mg of DMONs were dispersed into 100 mL of a mixture of ethanol and water (V _water_: V _ethanol_ = 1: 1), and 1 mL of APTES was added and continued to stir at 80 °C for 12 h. The reaction was carried out for 12 h, after which DMONs-NH_2_ was obtained by centrifugation using ethanol three times.

### Preparation of dsDNA

Double-stranded DNA molecules were generated by PCR amplification with DreamTaq polymerase using pcDNA3.1 as a template as described previously [37]. The length of the PCR products is 94 base pairs. The PCR products were purified using a GeneJET PCR purification kit.

Forward primer: 5’-CGATGTACGGGCCAGATATACG-3’;

Reverse primer: 5’-CATATATGGGCTATGAACTAATGACC-3’.

### dsDNA loading

DMONs-NH_2_ (Si: 100 ng/mL) was dispersed into 1 mL of dsDNA aqueous solution with different mass ratios of dsDNA to Si, and stirred overnight at room temperature, after which unloaded dsDNA was removed by centrifugation. The dsDNA concentration of the supernatant was detected by Nanodrop assay to calculate the loading capacity of dsDNA.

### In vitro dsDNA release

The in vitro dsDNA release profile of dsDNA@DMONs was studied in SBF (pH 7.4) with different GSH concentrations of 0, 5 and 10 mM at 37 °C with a shaking speed of 200 rpm. The concentration of dsDNA released in the supernatant at scheduled timepoints was determined using Nanodrop and followed by the replacement of buffer with fresh SBF with corresponding GSH concentrations.

### Material characterization

Transmission electron microscopy (TEM) images were acquired on a JEOL-2100F transmission electron microscope. Scanning electron microscopy (SEM) images and corresponding element mapping scanning were acquired on a field-emission Magellan 400 microscope (FEI Company, USA). The quantitative analysis of the Si element was determined by inductively coupled plasma mass spectrometry (ICP-MS, NexION 2000B, PerkinElmer, US). Confocal laser scanning microscopy (CLSM) images were acquired by FV1000 (SP8, Leica, US). Nitrogen adsorption–desorption isotherms were recorded at liquid nitrogen temperature with an ASAP 2020 adsorption analyzer (Micromeritics).

### IFN-I activity reporter assay

Murine IFN-Is activity was measured in RAW-Lucia ISG cells. Raw-Lucia ISG cells were seeded at a density of 30,000 cells per well in a 96-well flat bottom plate (Corning). After adhering to the plate, Raw-Lucia ISG cells were treated with different concentrations of dsDNA@DMONs for 24 h. Subsequently, 50 μL supernatant was transferred to a 96-well opaque white plate, and 50 μL QUANTI-Luc was added to detect luciferase activity.

### Agarose gel electrophoresis

After mixing the sample with 6 × loading dye, the sample was loaded into the well of 1% agarose gel containing Gelstain, and the gel was marked with a DNA marker. 1 × TAE buffer was used as the running buffer, and the electrophoresis was performed at a condition of 200 mA for 30 min using a nucleic acid electrophoresis apparatus. After electrophoresis, the gel was imaged using the ChemiDoc MP gel imaging system produced by Biorad.

### RNA extraction and quantitative real-time PCR

Total RNA was extracted from cells using the TRIzol Reagent and the Direct-zol RNA Miniprep Plus Kit according to the manufacturer's instructions. The RNA quality and quantity were evaluated using a microvolume spectrophotometer. Reverse-transcribed with the PrimeScriptTM RT reagent Kit with gDNA Eraser according to the manufacturer’s instructions. RT-qPCR was performed using SYBR Green and primer pairs designed to target *Ifnb*, *Cxcl10*, *Isg15*, *Tapbp*, *Tap1*, *Tap2*, *B2m*, *Psmb5* and *β-actin* (Primer sequences are listed in Supplementary Table 1). The relative mRNA expression levels were determined using the comparative C_T_ method, with normalization to the housekeeping gene β-actin, and the data was analyzed as fold induction compared to the control sample.

### Immunofluorescence analysis

Immunofluorescence and confocal analysis were performed as described before [38]. Sulfo-Cy5.5 NHS ester stock solution was prepared by adding 1 mL of DMSO to 1 mg of sulfo-Cy5.5 NHS ester powder. Then 100 μL of sulfo-Cy5.5 NHS ester stock solution was added to 1 mL of DMONs-NH_2_ and incubated overnight on a shaker protected from light. Afterward, the DMONs-Cy5.5 was obtained by centrifugation two times. B16-F10 or DC2.4 cells were cultured on glass-bottomed wells (150,680, Nunc) and treated with DMONs-NH_2_ for 30 min, 2 h or 6 h. After that, cells were fixed in 4% PFA in PBS for 10 min, washed twice with PBS, and then stained with DAPI for 10 min. At least eight representative images were taken for each sample using a Leica TCS SP8 confocal laser scanning microscope (CLSM).

### Protein extraction and immunoblotting

For western blotting experiments, cells were cultured with indicated treatments followed by being lysed with RIPA buffer supplemented with phosphatase and protease inhibitors and centrifuged for 15 min at 14,000 g in 4 °C. The protein concentration was determined by the PierceTM BCA Protein Assay Kit, and the protein samples were denatured using SDS-PAGE buffer by heating at 100 °C for 5 min. Subsequently, Equal protein amounts of samples were separated on 10% PAGE gels with a Multicolor Prestained Protein Ladder and then transferred to nitrocellulose membranes (Millipore) by the Trans-Blot Turbo Transfer System (Bio-Rad). The membranes were blocked in 5% BSA in TBST for 1 h at room temperature before incubation with primary antibodies at 4 °C overnight. The membranes were washed with TBST three times and incubated with HRP-conjugated secondary antibodies for 2 h at room temperature. Protein images were captured with the Tanon 5200 Series Fully Automated Chemiluminescence/Fluorescence Image Analysis System.

### In vivo tumor models

For in vivo tumor models, 1 × 10^6^ B16-F10 cells in 100 μL DMEM per mouse were used and injected subcutaneously into the flank of mice. The tumor volume was monitored every other day by measuring the length (a) and width (b), and the tumor volume was calculated to be 1/2a × b^2^. Mouse body weight was monitored during the therapeutic period. The mice in each group were intratumorally (*i.t.*) administrated with 50 µL/dose PBS, DMONs, dsDNA or dsDNA@DMONs for a total of four doses when the tumor volume was about 60 mm^3^. For the anti-PD-L1(Clone:10F.9G2) antibody, mice were administered with intraperitoneal injections of anti-PD-L1 antibody (q3d, 100 μg per mouse, a total of three doses). The study endpoint for maximum tumor volume was approximately 2000 mm^3^.

### Flow cytometry analysis of the tumor microenvironment

Tumor tissues were minced and enzymatically digested with 2 mg/mL collagenase I supplemented with 1 mg/mL hyaluronidase and 25 μg/mL DNase I for 30 min at 37 °C, to acquire a single-cell suspension. The dissociated cell suspensions were passed through a 70 μm filter, counted, and resuspended in 100 μL PBS to reach a cell density of 5 × 10^6^ cells per sample. Then the cells were blocked with anti-mouse CD16/32 antibody for 30 min. After washing with FACS buffer twice, cells were stained with Fixable Viability Stain for 15 min on ice in the dark. After washing, cells were stained with antibody mix (already titrated antibody concentrations) for 30 min on ice in the dark. After washing, cells were resuspended with 300 μL FACS buffer. For Ki67 and FOXP3 staining, cells were treated with eBioscience™ Foxp3 / Transcription Factor Staining Buffer Set by following the instructions and then stained with titrated antibodies. After washing, cells were resuspended with 300 μL FACS buffer. For intranuclear TNFα staining, cells were stimulated with Leukocyte Activation Cocktail according to the instructions before permeabilization with Fixation/Permeabilization Solution Kit, and then stained with titrated anti-TNFα antibody. Data were acquired with a FACSAriaTM III flow cytometer and analyzed by FlowJo software.

### H&E staining

Mice were euthanized at the final time point. The kidney, liver, spleen, heart and lung tissues were eviscerated, fixed in 4% formaldehyde and cut into 7-μm formalin-fixed paraffin-embedding (FFPE) slides. The FFPE slides were then stained with hematoxylin and eosin and then images were taken on an Olympus IX73 microscope.

### Supplementary Information

**Additional file 1.** Supplementary figures and tables.

## Data Availability

The data that support the findings of this study are available from the corresponding author upon reasonable request.
