# Supplementary material for: Tumor microenvironment-responsive DNA-based nanomedicine triggers innate sensing for enhanced immunotherapy
Source: J Nanobiotechnology. 2023 Oct 19;21:382. doi: 10.1186/s12951-023-02132-6 (PMC10585899; doi:10.1186/s12951-023-02132-6)
Supplement: Supplementary file 1 — Additional file 1. Supplementary figures and tables. [file 12951_2023_2132_MOESM1_ESM.docx]

Supporting Information

**Tumor microenvironment-responsive DNA-based nanomedicine triggers innate sensing for enhanced immunotherapy**

Jinyang Li^1,#^, Xiaoyu Han^1,#^, Shanshan Gao^1,#^, Yumeng Yan^1^, Xiaoguang Li^1,*^, Hui Wang^1,*^

*^1^ State Key Laboratory of Systems Medicine for Cancer, Center for Single-Cell Omics, School of Public Health, Shanghai Jiao Tong University School of Medicine, Shanghai 200025, China;*

*^#^These authors contributed equally.*

**Correspondence:** Email: huiwang@shsmu.edu.cn (H. Wang) or lixg@shsmu.edu.cn (X. Li).

**Supplementary Figures**

**
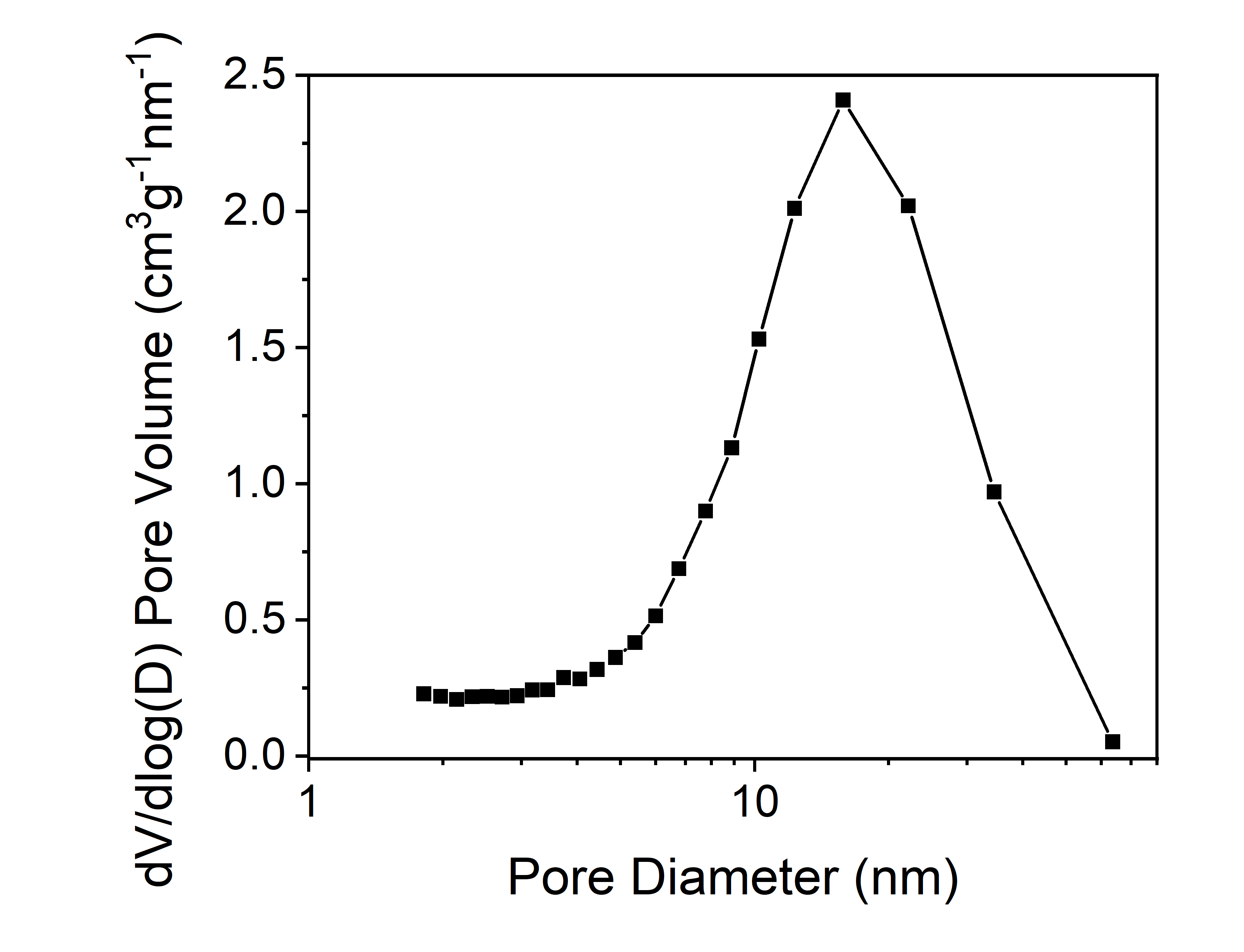
**

**Figure S1** The pore-size distribution of DMONs corresponds to N_2_ absorption-desorption isotherm.

**
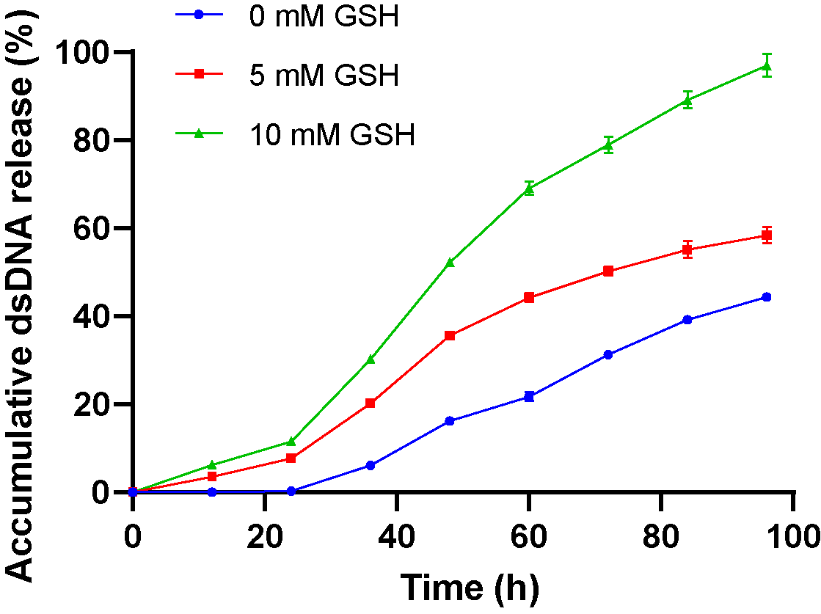
**

**Figure S2** Accumulative dsDNA release profiles of dsDNA@DMONs in SBF with various GSH concentrations (0 mM, 5 mM, 10 mM).

**
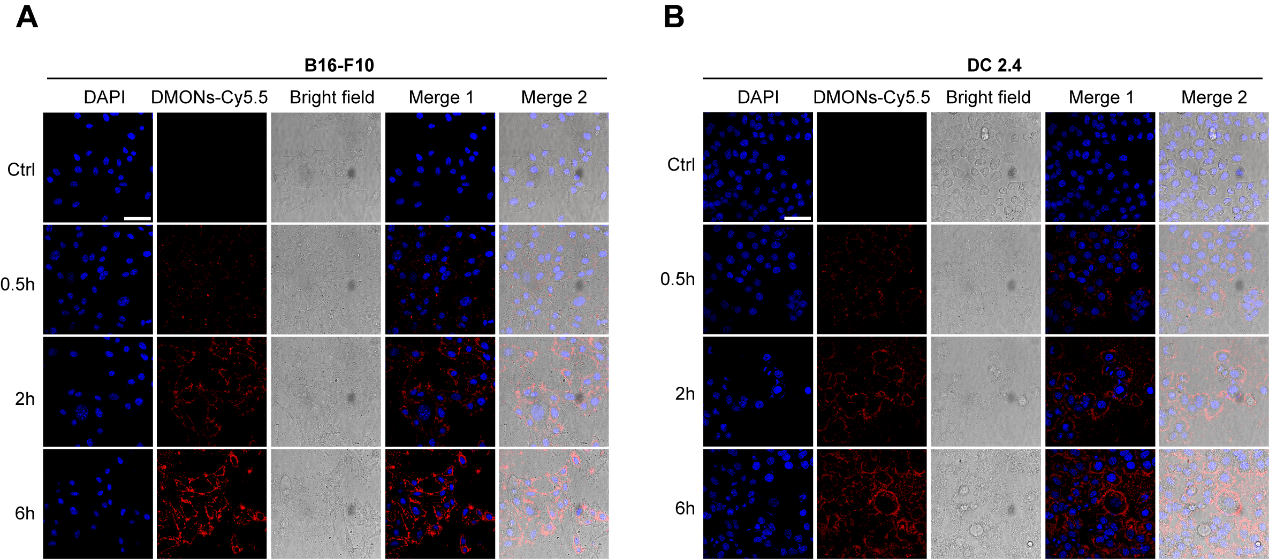
**

**Figure S3** The representative CLSM images of (**A**) B16-F10 cells or (**B**) DC2.4 cells after being treated with DMONs-Cy5.5 for 0.5, 2, and 6 h, with nuclear stained by DAPI (blue) and DMONs labeled with cy5.5 (red). "Merge 1" is a combined image consisting of DAPI and DMONs. "Merge 2" is a composite of "Merge 1" with a bright field view. Scale bar, 50 μm, magnification × 63.


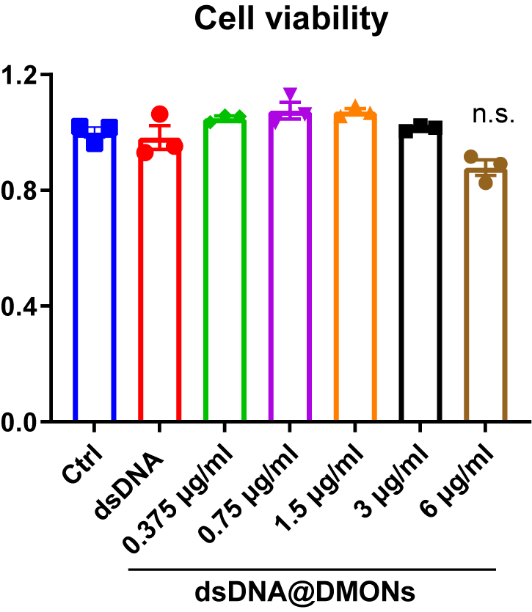


**Figure S4** Cell viability of RAW-Lucia ISG cells after being treated with DMONs for 24 hours. Data are shown as mean ± SEM (n≥3). P value was calculated by unpaired Student's t-test. (n.s., not significant, p>0.05).

**
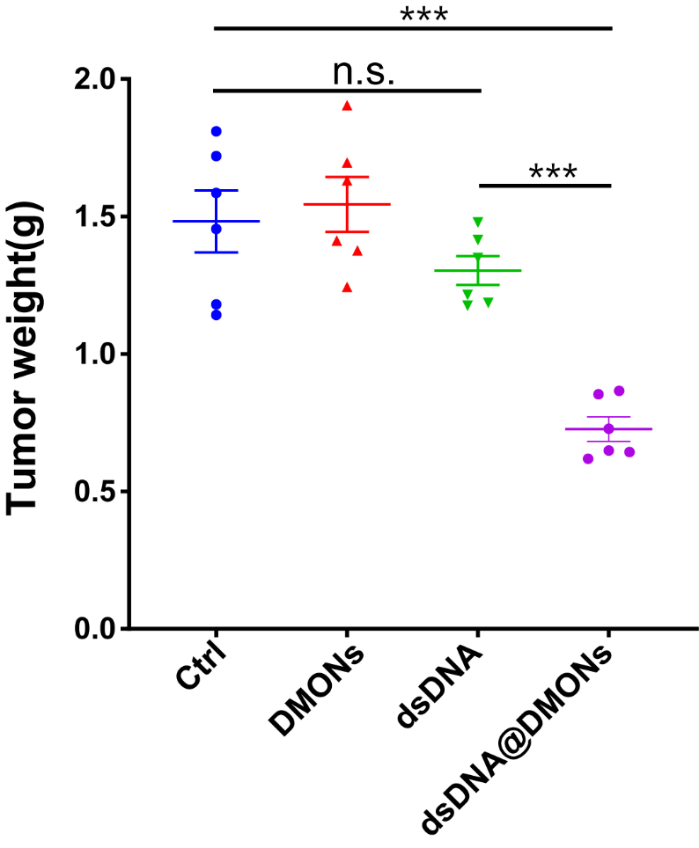
**

**Figure S5** Tumor weights of mice in each group**.** Data are shown as mean ± SEM (n=6). P value was calculated by unpaired Student's t-test. (n.s., not significant, p>0.05; ***p < 0.001).

**
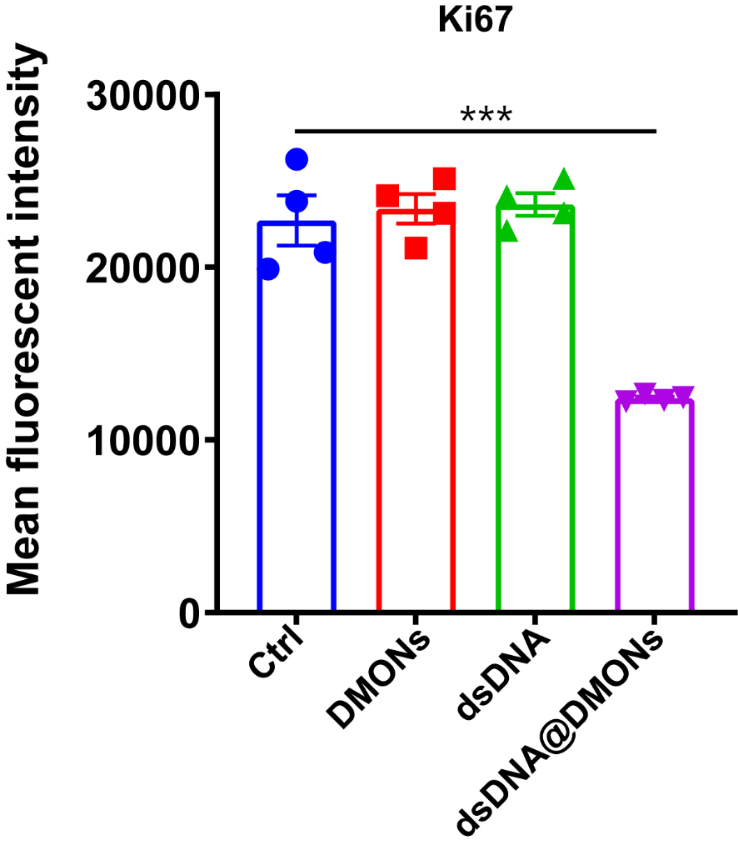
**

**Figure S6** Expression of Ki67 as detected by flow cytometry. Data are shown as mean ± SEM (n=4). P value was calculated by unpaired Student's t-test. (***p < 0.001).


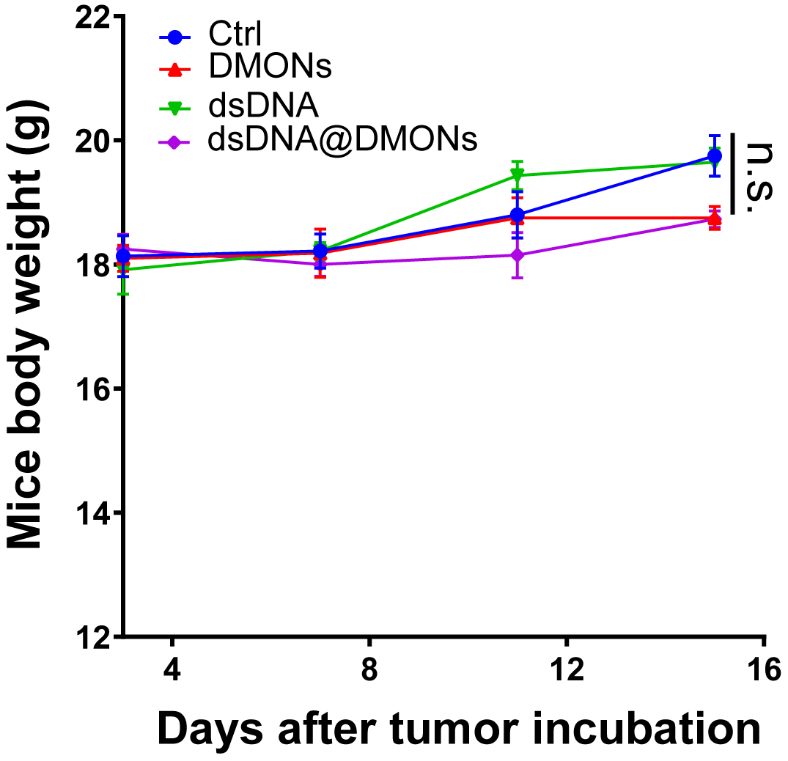


**Figure S7** Mice body weight monitored during the administration period. Data are shown as mean ± SEM (n=6). P value was calculated by two-way ANOVA (n.s., not significant, p>0.05)


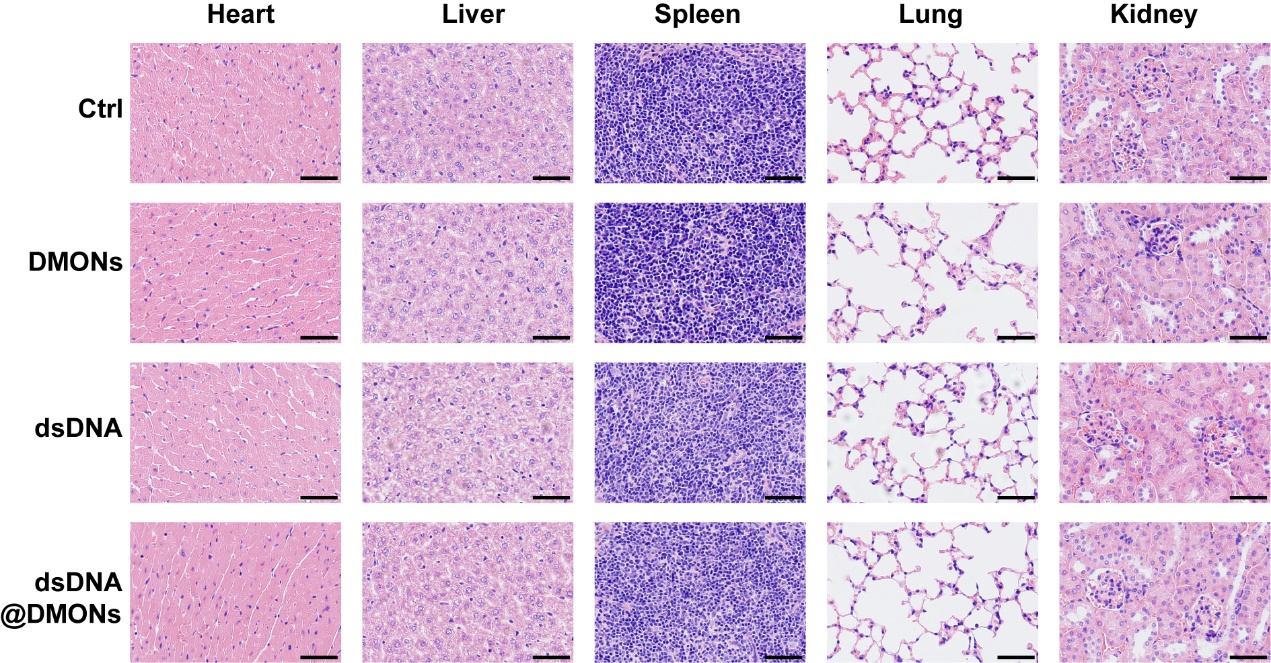


**Figure S8** H&E staining of major organs heart, liver, spleen, lungs, and kidneys from each group with a scale bar of 50 μm.


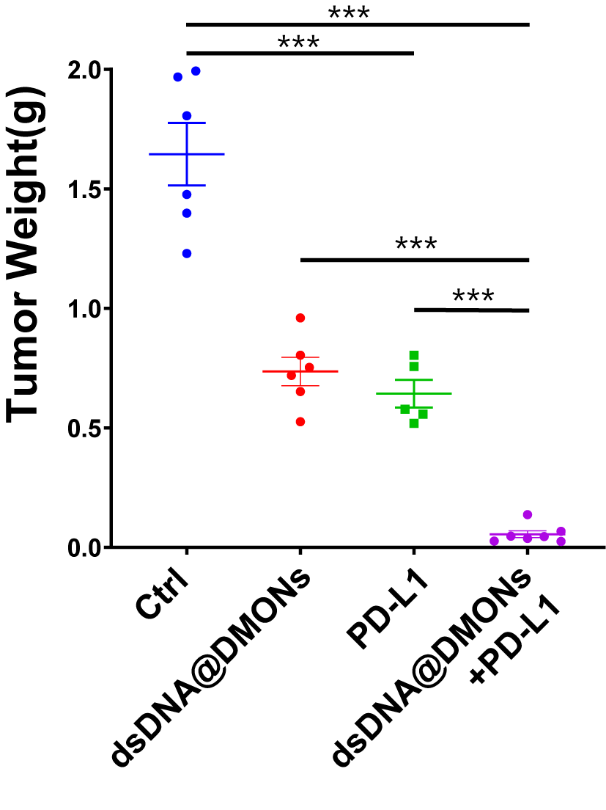


**Figure S9** Tumor weights of mice in each group**.** Data are shown as mean ± SEM (n≥5). P value was calculated by unpaired Student's t-test. (***p < 0.001)


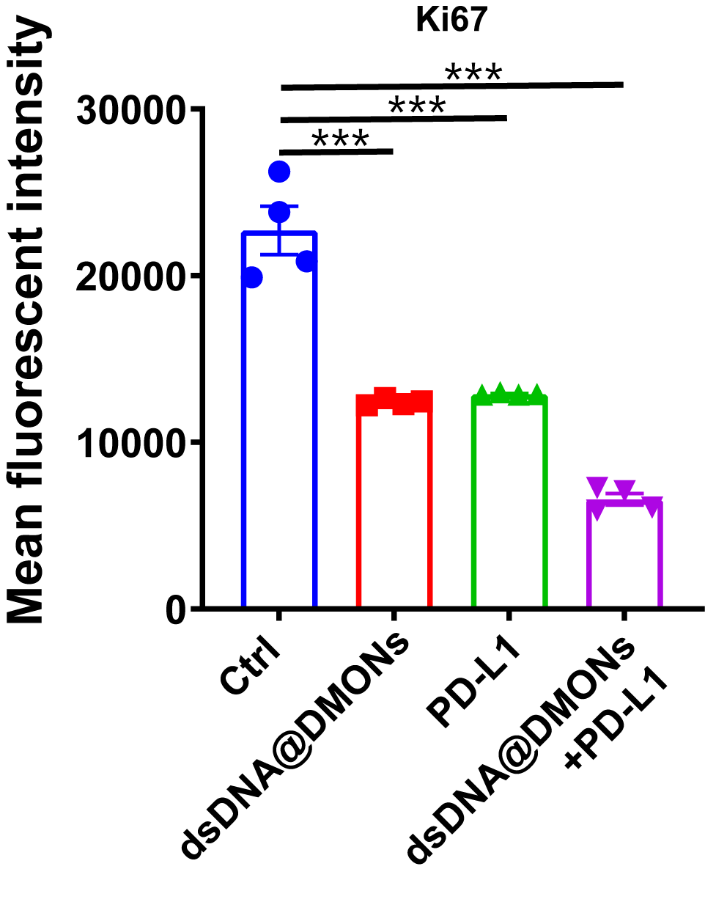


**Figure S10** Expression of Ki67 as detected by flow cytometry. Data are shown as mean ± SEM (n=4). P value was calculated by unpaired Student's t-test. (***p < 0.001)


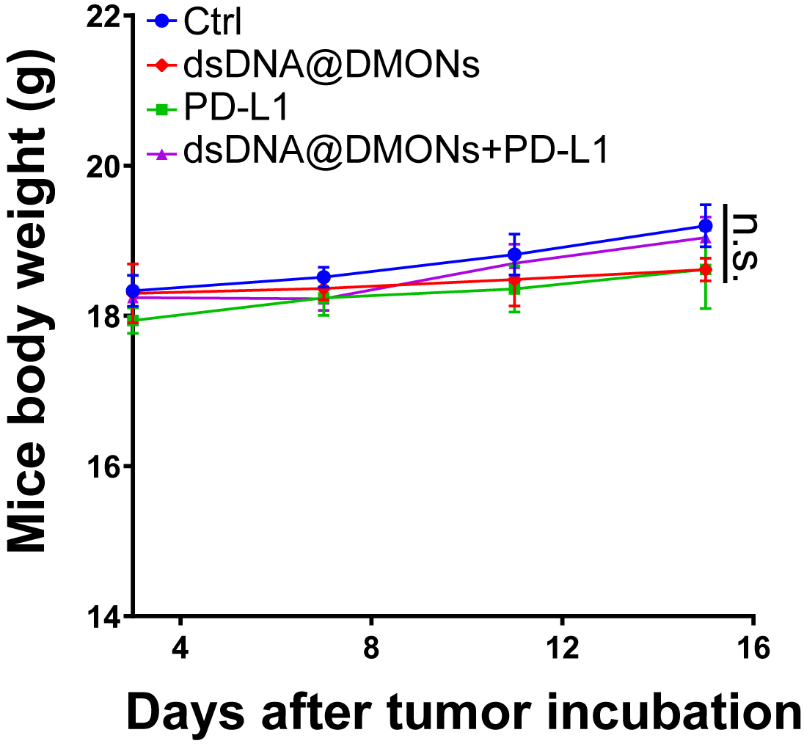


**Figure S11** Mice body weight monitored during the administration period. Data are shown as mean ± SEM (n≥5). P value was calculated by two-way ANOVA (n.s., not significant, p>0.05).


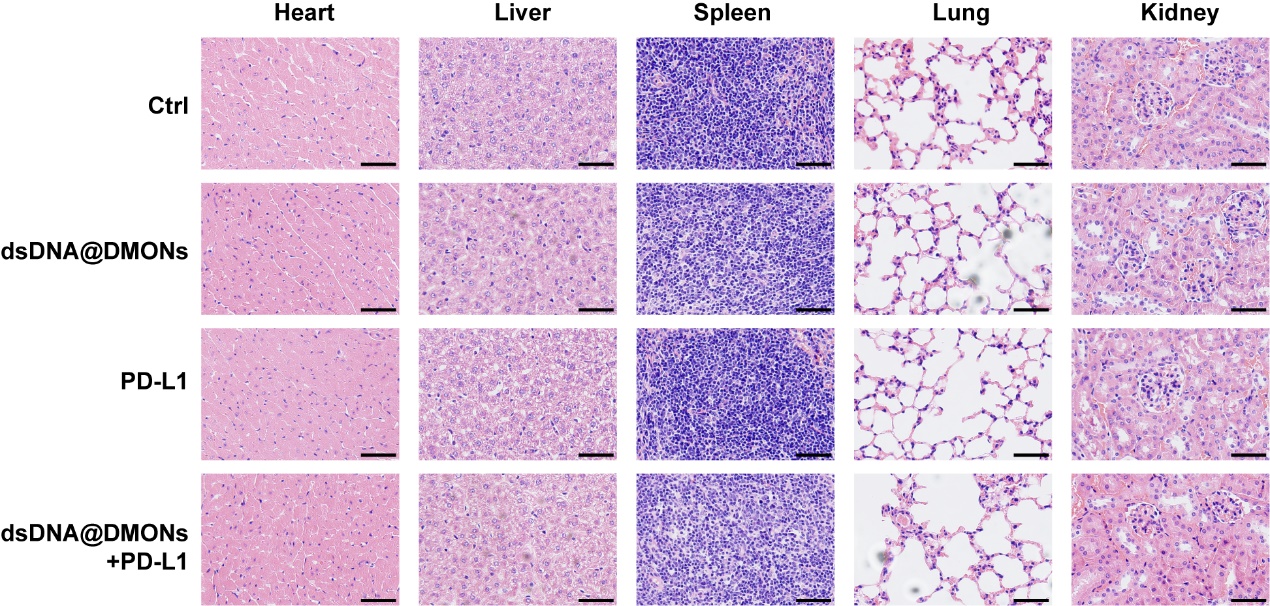
 **Figure S12** After euthanasia of mice, representative visual fields of HE staining of major organs heart, liver, spleen, lungs, and kidneys were taken from each group with a scale bar of 50 μm.

**Table 1 Primers for qRT-PCR**

| **Species** | **Gene** | **Forward sequence** | **Reverse sequence** |
| --- | --- | --- | --- |
| Mouse | *Ifnβ* | ATGAGTGGTGGTTGCAGGC | TGACCTTTCAAATGCAGTAGATTCA |
|  | *Cxcl 10* | GGAGTGAAGCCACGCACAC | ATGGAGAGAGGCTCTCTGCTGT |
|  | *Isg 15* | GAGCTAGAGCCTGCAGCAAT | TCACGGACACCAGGAAATCG |
|  | *L32* | TTAAGCGAAACTGGCGGAAAC | TTGTTGCTCCCATAACCGATG |
|  | *β-actin* | AGATCAAGATCATTGCTCCTCCT | ACGCAGCTCAGTAACAGTCC |
|  | *B2m* | TGGTCTTTCTGGTGCTTGTC | GGGTGGAACTGTGTTACGTAG |
|  | *Psmb5* | CGAATCGAAATGCTTCACGG | CAGAAGGTACGGGTTGATCTC |
|  | *Tap1* | CTCTTGGTGTTCATGTTTTGGG | CGTGGACTTTGCTAGAGACTC |
|  | *Tap2* | TCGTGTAATTGACATCCTGGG | CTGGACATGGTGAAGAGGAAG |
|  | *Tapbp* | AGCAGCATGGAGTTCACTATG | AGGAGAAAAGCAGACAGGAAC |
|  | *Pd-l1* | GCTCCAAAGGACTTGTACGTG | TGATCTGAAGGGCAGCATTTC |
| Human | *IFNβ* | AGTAGGCGACACTGTTCGTG | AGCCTCCCATTCAATTGCCA |
|  | *CXCL10* | CCACGTGTTGAGATCATTGCT | TGCATCGATTTTGCTCCCCT |
|  | *ISG15* | ACAGCCATGGGCTGGGAC | GGTTCGTCGCATTTGTCCAC |
|  | *β-ACTIN* | CTCGCCTTTGCCGATCC | TCTCCATGTCGTCCCAGTTG |

[1] LUECKE S, HOLLEUFER A, CHRISTENSEN M H, et al. cGAS is activated by DNA in a length-dependent manner [J]. EMBO Rep, 2017, 18(10): 1707-15.

[2] DAEMEN S, CHAN M M, SCHILLING J D. Comprehensive analysis of liver macrophage composition by flow cytometry and immunofluorescence in murine NASH [J]. STAR Protocols, 2021, 2(2): 100511.
